# Unraveling the hidden world: Variability and complexity of holopelagic *Sargassum* biofilms

**DOI:** 10.1016/j.bioflm.2026.100362

**Published:** 2026-04-16

**Authors:** Zujaila Nohemy Qui-Minet, Christine Paillard, Valérie Michotey, Solène Connan, Philippe Elíes, Valérie Stiger-Pouvreau

**Affiliations:** a Univ Brest, CNRS, IRD, Ifremer, LEMAR, Plouzané, F-29280, France; b Aix Marseille Univ, Université de Toulon, CNRS, IRD, MIO, Marseille, France

**Keywords:** Holopelagic *Sargassum* spp., *Sargassum* strandings, Macroalgal biofilm, Holobiont, Biofilm architecture, Scanning electron microscopy, Calcium phosphate (Ca–P–O) crystals

## Abstract

Holopelagic *Sargassum spp.* —including *S. natans* var. *natans* (SNN), *S. natans* var. *wingei* (SNW), and *S. fluitan*s var. *fluitans* (SFF)—have become a major ecological and socio-economic concern due to recurrent mass strandings in the Caribbean. This study aims to complement the growing knowledge on the holopelagic *Sargassum* holobiont by incorporating the three-dimensional organization of its biofilms. Here, we provide the first detailed characterization of holopelagic *Sargassum* biofilms using scanning electron microscopy (SEM) combined with bacterial abundance measurements. Samples were collected from nearshore waters and stranded mats in Guadeloupe. We assessed biofilm elemental composition, maturity, and surface coverage, along with the presence of diatoms and superficial filamentous bacterial coverage (SFBC). Here, we report for the first time in macroalgal biofilms the occurrence of biofilm-coated diatom units (BCDU) with protrusions, as well as Ca–P–O crystals.

Biofilm structure and composition varied among taxa, thallus compartments, and environmental contexts. SNN exhibited the lowest surface roughness, the weakest biofilm development in nearshore conditions, and the lowest abundances of Ca–P–O crystals. In contrast, SFF consistently displayed high densities of crystals, diatoms, and BCDU with protrusions, indicating greater microbial and morphological complexity, while SNW maintained a stable biofilm architecture largely independent of thallus part and environment. Stranding increased bacterial abundance across all taxa and enhanced diatom colonization in SNW and SFF. Across taxa, axes were least affected, whereas fronds, particularly in SNN,were most strongly impacted. SFBC, highest in SNN, was negatively correlated with crystal abundance, which was highest in SFF and SNW.

Overall, these findings provide a three-dimensional perspective on intra-thallus and interspecific variation in *Sargassum* biofilms and demonstrate how interactions among host surface traits, microbial components, and environmental context shape biofilm structure, stability, and potentially host resilience.

## Highlights

• Biofilm traits vary across *Sargassum* thallus parts and morphotypes, potentially affecting resilience.
• Surface roughness differs within and between morphotypes, affecting biofilm development.
• First detection of Ca-P-O crystals in macroalgal biofilms, negatively linked to superficial filamentous bacteria presence.
• Fronds are most prone to change upon stranding; bacterial density rises, and filamentous bacteria coverage differ by taxa.
• Biofilm-coated diatoms with protrusions seen in SFF and SNN but with an unclear ecological role.

## 1 Introduction

Macroalgae, as primary producers, play a crucial role in marine ecosystems [1]. These multicellular photosynthetic organisms contribute significantly to coastal primary production, shape intertidal environments, and provide essential habitats and refuges for numerous marine species. As such, they are essential components of coastal ecosystems. Over the last years, massive macroalgal bloom and strandings have increased both in frequency and magnitude [2]. Notably, since 2011 the Great Atlantic *Sargassum* Belt has been detected in the entire tropical Atlantic, from West Africa to the Caribbean Sea and Gulf of Mexico, characterized by vast floating rafts of holopelagic *Sargassum* that have begun reaching Caribbean coastlines. These drifting aggregations primarily consist of three taxa or morphotypes: *Sargassum natans* var. *natans* SNN (known before as *S. natans* I), *S. natans* var. *wingei* SNW (known before as *S. natans* VIII) and *S. fluitans* var. *fluitans* SFF (known before as *S. fluitans* III) [3]. While the lifespan of holopelagic *Sargassum* thalli under favorable conditions is still unclear, rapid decay typically occurs after they reach coastal waters and become stranded along shorelines [4]. The further degradation of these massive strandings has led to significant ecological damage, as well as negative impacts on human health and local economies [[5], [6], [7]].

Over recent decades, research has highlighted the close interdependence between multicellular organisms and their associated microbiomes, leading to the holobiont concept [8], which views the host and its microbiome as a single evolutionary and functional unit [9]. Marine macroalgae exemplify this relationship, as their organic-rich surfaces support diverse and dense microbial biofilms [10], dominated by bacteria, vastly outnumbering eukaryotic microorganisms, such as diatoms and flagellates, at an estimated ratio of 640:4:1, respectively [11]. Fungi, protozoa, and viruses also contribute to the macroalgal microbiome [8,12]. These biofilms are highly dynamic [10] forming through successive physical, chemical, and biological interactions [13], initiated by extracellular polymeric substances (EPS) and transparent exopolymer particles (TEPs), and developed into complex, multilayered matrices that structure microbial interactions. This matrix comprises a complex mixture of water, polysaccharides, proteins, lipids, metabolites, extracellular DNA, signalling molecules, waste products and detritus [14,15]. Within this framework, bacteria play central mutualistic roles by utilizing algal-derived carbon and oxygen [13], while supplying essential minerals, vitamins, and growth factors that support macroalgal growth, development and reproduction, while also contributing to pollutant scavenging [12,[16], [17], [18]]. Biofilm structure and behavior are further regulated by physico-chemical parameters [8,19], as well as biochemical mechanisms including adhesins, quorum sensing and secondary messengers [19]. Advances in microscopy and molecular tools have revealed biofilms as structurally complex three-dimensional systems, whose spatial organization governs nutrient diffusion, microbial interactions, and overall biofilm function [20,21]. In this respect, five stages of biofilm maturity are recognized: (1) initial attachment, (2) formation of monolayer and production of matrix (irreversible attachment), (3) microcolony formation and multilayer (maturation I), (4) matrix formation, with characteristic mushroom-shaped structure composed of polysaccharides (maturation II) and at last, (5) dispersion [22].

**Fig. 1:**
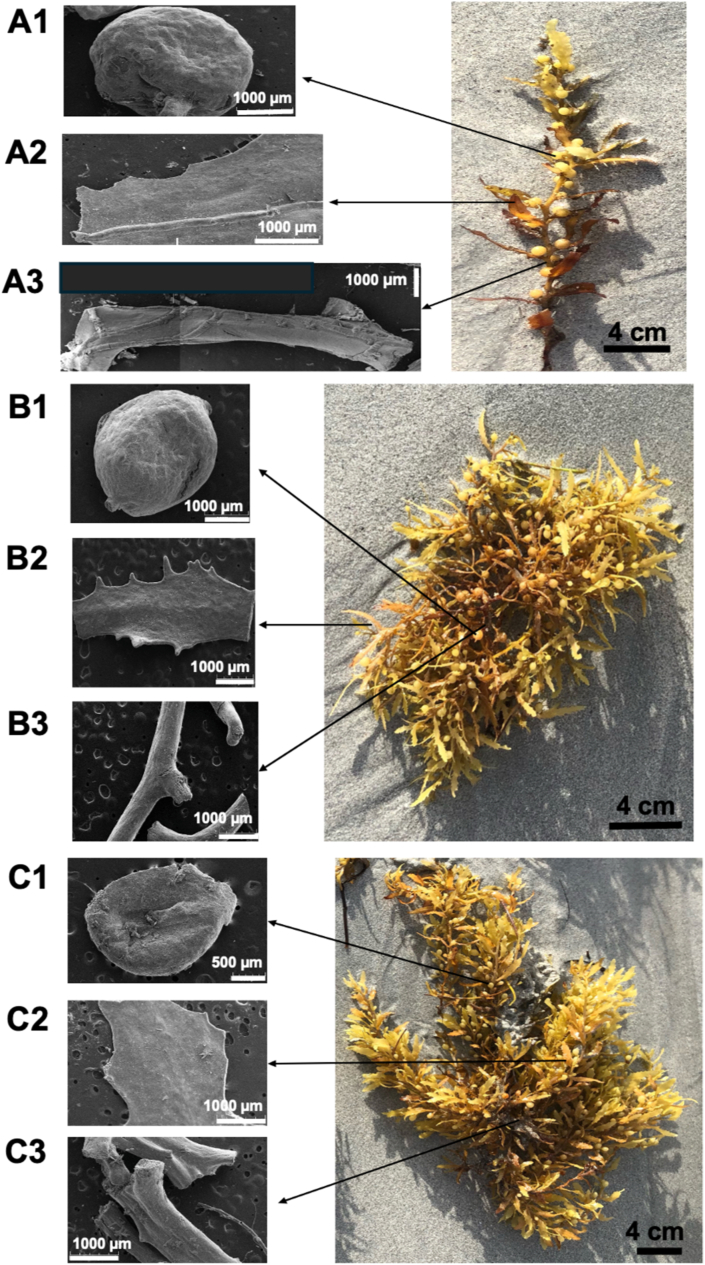
**SEM mages of the three holopelagic *Sargassum* taxa, showing the distinct parts of their thallus** — floating vesicles (A1, B1, C1), fronds (A2, B2, C2), and axes (A3, B3, C3) — captured using a scanning electron microscope at a magnification of 1000x. Panels: A = SNW (*S. natans* var. *wingei*), B=SNN (*S. natans* var. *natans*), and C=SFF (*S. fluitans* var. *fluitans*). Labels: 1 = floating vesicles, 2 = fronds, and 3 = axes. Red arrows show the peduncles attached to floating vesicles. (For interpretation of the references to colour in this figure legend, the reader is referred to the Web version of this article.)

Within this context, the objective of this study was to characterize how biofilms associated with the three holopelagic *Sargassum* taxa involved in massive Caribbean strandings i.e. SNN, SNW and SFF, are affected by stranding, in comparison with nearshore specimens. These holopelagic varieties have the complex thallus structure typical of *Sargassum,* except for the absence of a holdfast. The thallus comprises an axis, fronds, and floating vesicles (Stiger-Pouvreau et al., [23]; Guiry, [24]). Floating vesicles are peduncled and present on the axis (main axis or ramifications; Fig. 1). These taxa exhibit notable differences in biochemical and tissue composition, as well as in growth patterns [[25], [26], [27], [28], [29], [30]]. They also display inter-annual variability in biomass, with one taxon typically dominating Caribbean strandings each year [[31], [32], [33]]. This work compares nearshore and stranded samples to investigate biofilm changes during decay. Specifically, we address the following questions: (a) in morphologically complex seaweeds, how does biofilm composition vary among thallus parts; (b) which taxa are more resistant to decay?; and (c) which thallus components are the first to undergo decay? We hypothesize there are: a) intra-taxon differences in biofilm characteristics **across thallus parts**, reflecting variation in biofilm development across different parts of their thallus; b) **location-driven differences** in biofilm characteristics **among taxa**, highlighting distinct responses to environmental change; and c) spatial differences of biofilm **within thallus parts between nearshore and stranded** specimens, reflecting variations in resilience through different locations within the same taxa.

## 2 Material and methods

### 2.1 Sample collection

Samples were collected around Guadeloupe Island (French overseas territory) at two distinct stations: stranded on the coastline (Viard Beach “Petit Bourg” 16°09.816 N; 61°35.060 W) and nearby in the nearshore (16°11.061 N; 61°25.523 W), 2.70 km from the nearest coast and 17.13 km from the stranded station. Samples were collected in December 2020 and gently rinsed with filtered seawater to remove sand and debris.

### 2.2 Microscopic analyses

#### 2.2.1 Bacterial counts using Vivatome analysis (fronds and peduncles of floating vesicles)

For bacterial counts using Vivatome analysis, specimens were preserved in 3% formaldehyde in filtered seawater immediately after collection at the two stations (stranded and nearshore). Bacterial counts were performed on both fronds, and peduncles of floating vesicles. The samples (thallus fragments consisting of fronds and peduncles of floating vesicles attached to axes) were preserved in 2.5% glutaraldehyde in sterile filtered seawater. Each sample was stained with 2% DAPI (4′,6-diamidino-2-phenylindole dihydrochloride) (Abcam) for approximately 10 min before being observed. The samples were analyzed using a Zeiss VivaTome, which allows a sample to be observed while taking several optical section photographs and assembling them using the “Z-stack” function to obtain a 3D rendering and visualize the biofilm in its entirety. Blue (Ex: λ = 406/15 nm; Em: λ = 457/50 nm) and red (Ex: λ = 575/25 nm; Em: λ = 628/40 nm) filters were used to visualize the fluorescence of bacterial DNA, as well as the autofluorescence of epiphytes present on the surface of the brown macroalga. For each taxa (SFF, SNN, and SNW) and station (nearshore and stranded), 15 images of fronds (40x) and 5 images of peduncles from floating vesicles (63x) were acquired for each of the 3 taxa, resulting in 45 fronds images and 15 peduncle images per taxon and station. The images obtained were then analyzed using ImageJ software to quantify the bacteria abundance. Each image represents a total surface area of 2.0 × 10^−2^ mm^2^ for a frond and 7.1 × 10^−3^ mm^2^ for a peduncle of a floating vesicle. The number of bacteria was reported in bacteria per mm^2^.

#### 2.2.2 Acquisition of SEM images (axes, fronds and floating vesicles) for biofilm characterisation

For scanning electron microscopy (SEM) analysis of axes, fronds, and floating vesicles, specimens were fixed *in situ* in a 2.5% glutaraldehyde solution prepared in filtered seawater and incubated for 24 h. Samples were rinsed three times in 0.2 M phosphate buffer pH = 7.2. Once in the lab, samples underwent progressive dehydration in different ethanol baths from baths in 50° ethanol to final baths in absolute ethanol [34]. Then the samples were exposed to a solution of hexamethyldisilazane (HMDS), gradually decreasing the alcohol:HMDS ratio of: 3:1 (15 min), 1:1 (15 min), 1:33 (15 min), 0:1 (2 × 15 min). Samples were glued to studs with silver lacquer (Silver Conductive Adhesive 504) and metallized using a sputter coater (5 nm Au–Pd). Each part was observed using the Hitachi S–3200 N scanning electron microscope at UBO's joint electron microscopy Common Service.

Images and compositional analysis of all samples were acquired with a HITACHI S–3200 N scanning electron microscope (SEM) equipped with an energy-dispersive X-ray (EDX). In total, 1408 images were acquired, covering all treatments, thallus parts of each taxon, both locations, and multiple magnifications. This dataset supported qualitative analyses of biofilm elemental composition, surface structures, and macroalgal surface roughness, as well as quantitative analyses of biofilm metrics from axes, fronds, and floating vesicles.

#### 2.2.3 Elemental composition of biofilm and surface structures

Surface microanalysis was performed using SEM to characterize the elemental composition of holopelagic *Sargassum* biofilms. Elemental identification was conducted using spectral measurements from images captured at 1000x magnification, with samples irradiated by a 15 keV electron beam, corresponding to an acceleration voltage of 15 kV. Element detection relies on backscattered electron yield, which depends on the average atomic number, from which heavier elements possess a more positive charge and thus, a higher yield [35]. This approach enabled qualitative characterization of elements present at the biofilm surface (the elements present at a 4-μm interface), as well as within associated structures such as crystals and biofilm-coated diatom units with protrusions (BCDU with protrusions). Due to the uneven surface of seaweed tissues, this method does not provide accurate quantitative estimates of elemental proportions; but gives qualitative data on the elements present on the macroalgal surface. Therefore, subsequent analyses focus solely on the presence or absence of elements associated with specific structures: crystals and BCDU with protrusions.

#### 2.2.4 Surface roughness of each part of the SNN, SNW, and SFF thalli

To estimate and compare surface roughness among holopelagic *Sargassum* taxa, scanning electron microscopy (SEM) images of different thallus parts were acquired at 2500 × and 1000 × magnification. All images were imported into Fiji (ImageJ, version 2.16.0/1.54p; National Institutes of Health, USA) and converted to 8-bit grayscale to standardize pixel depth. Surface texture was quantified using the Gray Level Co-occurrence Matrix (GLCM) method [36] implemented in the Texture Analyzer plugin. For each image, GLCM features were calculated at a pixel offset of 1 (step size = 1 pixel) in four orientations (0°, 45°, 90°, and 135°). The following parameters were extracted: (i) Angular Second Moment (ASM, or Energy), which quantifies the uniformity of pixel intensity distribution; (ii) Contrast, which measures local intensity variation between neighboring pixels; (iii) Correlation, which indicates the degree of linear dependency between pixel intensities; (iv) Inverse Difference Moment (IDM, or Homogeneity), which reflects similarity among neighboring gray levels; and (v) Entropy, which describes the randomness of the gray-level distribution. Values obtained from the four orientations were averaged to produce orientation-independent descriptors of surface texture. In this framework, higher contrast and entropy values indicate greater surface roughness, whereas higher ASM, correlation, and IDM values correspond to smoother surfaces. Nevertheless, surface roughness is inherently a three-dimensional parameter, typically quantified using height variations (Ra, Rq, etc.). A two-dimensional image captures only surface and not volume. Nevertheless, our images reveal clear differences in surface texture among thallus parts and taxa (Fig. 6), consistent with our analytical observations.

#### 2.2.5 Biofilm complexity metrics

**Fig. 2:**
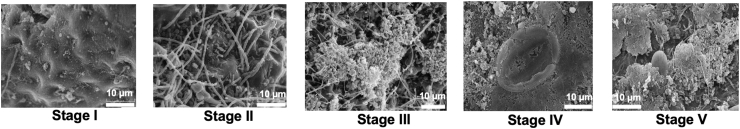
**SEM images from seaweed samples displaying the different stades of biofilm development.** Stage I: initial attachment, Stage II: formation of monolayer and production of matrix (irreversible attachment), Stage III: microcolony formation and multilayer (maturation I), Stage IV: matrix formation, with characteristic mushroom-shaped structure composed of polysaccharides (maturation II) and at last, Stage V: dispersion.
b) *Biofilm and superficial filamentous bacterial coverage (SFBC).* Biofilm and SFBC were quantified using ImageJ2 software (version 2.3.0/1.53q). Images were acquired at 2500 × magnification from 4 biological replicates. For each image, we measured the total surface area and the area covered by biofilm or superficial filamentous bacteria; we then calculated the percentage of the seaweed surface covered by the biofilm and superficial filamentous bacteria.
c) *Diatoms, crystals and BCDU with protrusions.* The presence of diatoms, crystals and BCDU with protrusions was not assessed based on surface coverage. Instead, their abundance was quantified as the number per surface, using images taken at a 200 × magnification (n = 4).

Along with our analysis, we were interested by three different metrics to characterize the biofilm of each part of the thallus (axis, frond and floating vesicle) of each taxon (SNN, SNW and SFF) at each location (stranded and nearshore):

a) *Biofilm development.* Depending on their maturity, biofilms can be classified in the 5 stages previously described [15] (Fig. 2). According to this classification and in order to characterize the *Sargassum* spp. biofilms, biofilm maturity was evaluated from 4 biological replicates of each thallus part at a magnification of 2500x. Each stage of biofilm development was assigned a numerical value (e.g., 1 for the initial stage and 5 for the dispersion stage). When an image exhibited biofilm heterogeneity indicating the presence of two distinct maturity stages, an average value was calculated to represent the combined maturity level observed in that image.

### 2.3 Statistical analyses

Statistical analyses were performed on the different measured variables using the open-source software RStudio version 4.3.1 (R Core Team, 2023). The normality and the homogeneity of variances were tested with the Shapiro Wilk's and the Levene's tests, respectively. Since the data were non-parametric, a Kruskal-Wallis (KW) test was performed to examine how biofilm characteristics were influenced by: (1) different parts of the thallus within the same variety (SNN, SNW, SFF) and location (stranded and nearshore stations), (2) location, and finally (3) the different parts of the thallus of the *Sargassum* varieties (floating vesicles, fronds, and axes). The Wilcoxon–Mann–Whitney test was used to compare bacterial abundance between fronds and peduncles of floating vesicles, among taxa, and between locations. When significant differences were observed, we performed Dunn's post-hoc test to identify the specific group pairs that differed. To evaluate the relationship between SFBC coverage and crystal abundance, we calculated Kendall's rank correlation coefficient (τ). Kendall's τ was chosen because the data contained tied values and did not meet the assumptions of normality required for parametric correlation tests. Significance was assessed using a two-sided test (*p* < 0.05).

## 3 Results

The results are organized into two main sections. The first section presents qualitative analyses, describing surface roughness and biofilm elemental composition across the three taxa, as well as the specific composition of crystals and BCDU with protrusions within the biofilm. The second section presents quantitative analyses, assessing biofilm development and coverage, the occurrence of diatoms, crystals, and BCDU with protrusions on axes, fronds, and floating vesicles, and bacterial abundances on fronds and peduncles of floating vesicles.

### 3.1 Qualitative analyses

#### *3.1.1* Sargassum *surface roughness*

**Fig. 3:**
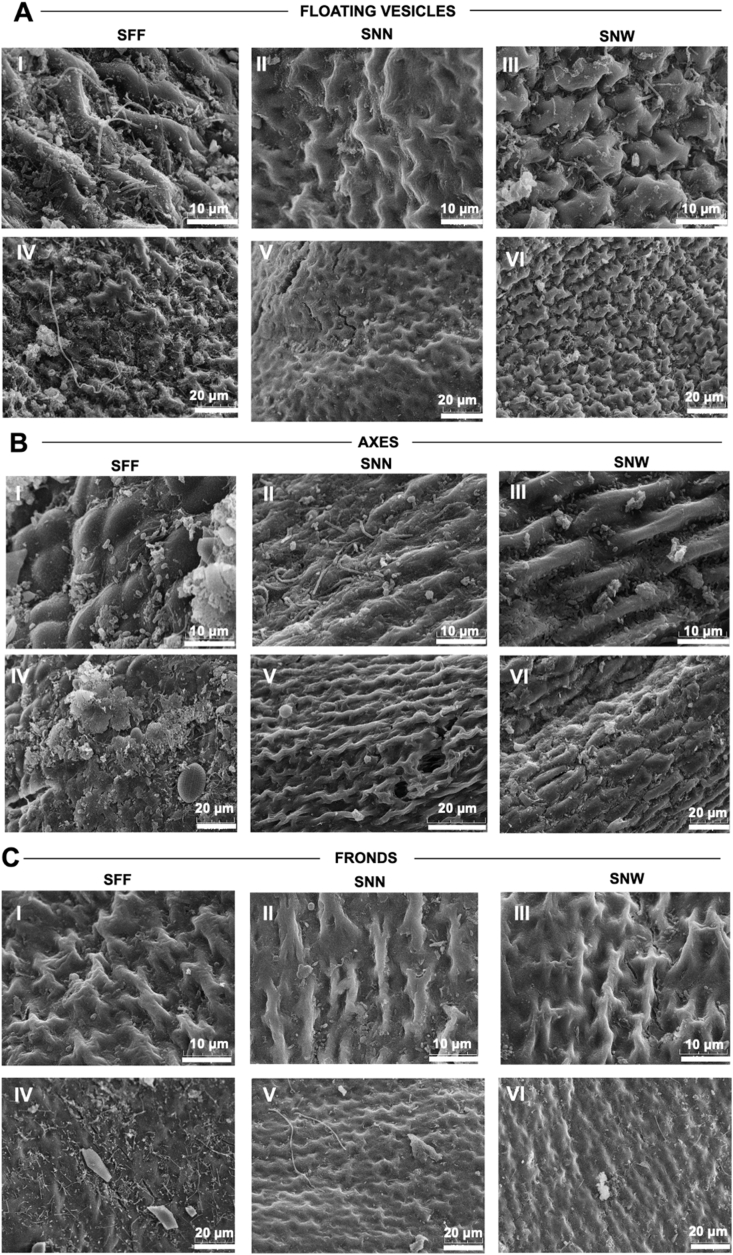
**SEM images of (A) floating vesicles, (B) axes, and (C) fronds of *Sargassum natans* var. *natans* SNN** (I, IV), *Sargassum fluitans* var. *fluitans* SFF (II, V) and *Sargassum natans* var. *fluitans* SNW (III, VI). Bars = 10 μm (2500x magnification) for I, II, III; and 20 μm (1000x magnification) for IV, V et VI.

Estimations of relative surface roughness differed significantly among taxa and among thallus parts in SNN (Kruskal–Wallis, *p* < 0.0001; Table S1), as also supported by qualitative observations of 2D surface texture features, including the presence of ridges, grooves, pores, and overall heterogeneity (Fig. 3). Overall, SNN exhibited smoother surfaces than SNW and SFF, as reflected by lower roughness-related parameter values across thallus parts. Notably, SNN was the only taxon showing significant intra-thallus variation in surface roughness, indicating marked differences among axes, fronds and floating vesicles. Parameters associated with rougher surfaces (contrast and entropy) were significantly higher for SFF-axes compared to SNN-axes (*p* < 0.05) and for SFF-vesicles (*p* < 0.01) and SNW-vesicles compared to SNN-vesicles (*p* < 0.01; Dunn's tests; Table S1). In contrast, no significant differences were observed among SFF, SNN and SNW fronds (*p* < 0.01). Conversely, parameters linked to smoother surfaces (ASM, correlation, and IDM) generally exhibited higher values for SNN than for SNW and SFF (Dunn's tests, *p* < 0.05–0.001; Table S1), reinforcing the overall pattern of lower surface roughness in SNN. Within SNN, contrast and entropy did not differ among thallus parts. However, ASM was significantly lower in fronds compared to axes, and IDM was significantly lower in vesicles than in fronds, indicating reduced smoothness in SNN-fronds, relative to other SNN-thallus parts. Together, these localized differences point to greater surface heterogeneity within SNN compared with SFF and SNW.

#### *3.1.2* *Identification of microbial elements on holopelagic* Sargassum *biofilms*

Using microscopic approaches, we identified a diverse assemblage of microbial organisms at the surface of the biofilms, including bacteria with multiple morphologies and diatoms. Diatoms were also observed inside protuberant structures, which we termed biofilm-coated diatom units with protrusions (BCDU with protrusions). Across all taxa and conditions, adnate diatoms, i.e. *Cocconeis* spp., were the dominant forms (Fig. S1). In addition, soft, pendant lateral branches (∼20 μm wide and >1 mm long) arising from the main axes and densely covered with adnate diatoms were observed in SFF and SNN (Fig. S2), Although SEM does not allow precise taxonomic identification of bacteria, filamentous morphotypes consistent with cyanobacteria or heterotrophic bacteria were observed. Other bacterial morphologies were also detected across the three taxa, including curved-rod shaped cells consistent with *Vibrio* spp. (Fig. S3).

#### 3.1.3 Elemental composition of biofilm and surface structures (axes, fronds and floating vesicles)

##### 3.1.3.1 Biofilm elemental composition

Major elements commonly found on living organisms, and already identified on seaweed, including C, H, O, N, P, and S; macro-minerals such as Ca, Mg, Cl and Na; trace elements including Fe, Mn, Cu, Co, Se, I, B, and Ni; as well as potential contaminants like As and Al, were all tested for their presence on the surface of holopelagic *Sargassum* [37]. The identified elements included C, O, Na, Mg, Al, Si, P, S, K, Ca, and As (Fig. S4), Cl was also identified, but only in SFF stranded samples (Fig. S4). The peaks of energy of Mg (1.25 keV) and As (1.28 keV) are small and overlap each other (Fig. S5). As noted in the Materials and Methods section, the uneven surface of the macroalgal tissues, even the fronds, prevented precise quantification of the proportion of each element. Consequently, it was not possible to assess significant differences in the elemental distribution among species or between different parts of the thallus.

##### 3.1.3.2 Crystals composition

**Fig. 4:**
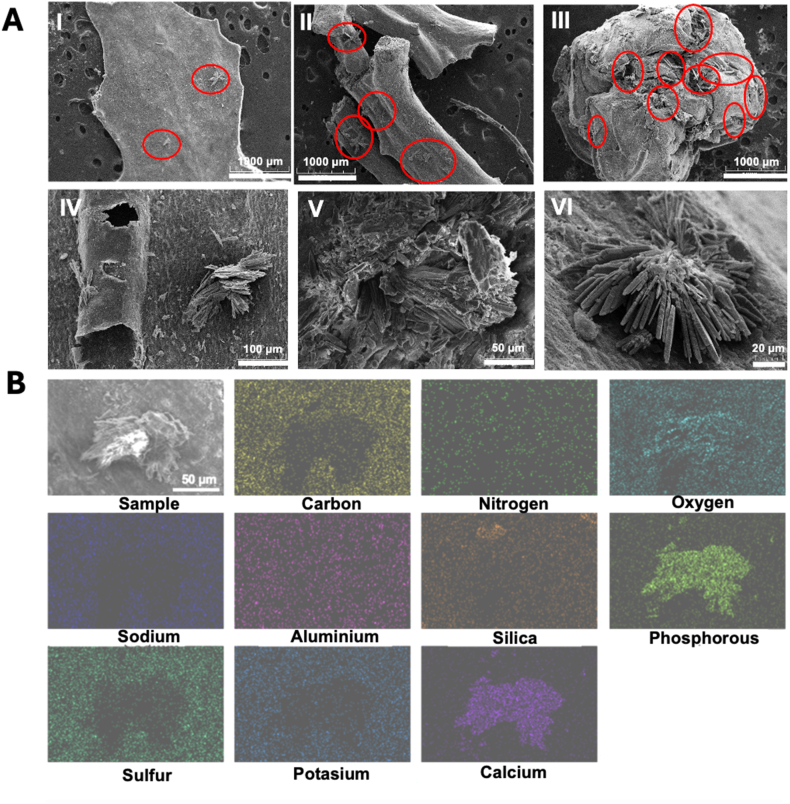
(**A**) **SEM images of crystalline structures identified at the surface of *Sargassum fluitans* var. *fluitans* SFF and *S. natans* var. *wegei* SNW** using a different magnification: I. SFF-fronds (20x magnification), II. SFF-axis (20x magnification), III. SNW-vesicles (50x magnification), IV. SNW-fronds (150x magnification) V. SNN-vesicles (500x magnification) VI. SFF-vesicles (1000x magnification). (**B**) **Elemental composition mapping of the crystals found at the surface of *Sargassum* samples**. Red circles in panels I–III indicate the crystals highlighted in the images. (For interpretation of the references to colour in this figure legend, the reader is referred to the Web version of this article.)

Crystalline structures were observed on the surface of *Sargassum* taxa (Fig. 4A & Fig. S6), except on SNN-fronds (both stranded and nearshore samples) and SNN-vesicles (stranded samples). SEM–EDS analysis detected Ca, P, and O in these structures (Fig. 4B), consistent with calcium phosphate (Ca–P–O), although further analysis is required to verify the stocihiometric formula. In addition to these elements, N and Al were detected across the analyzed image, without clear distinction between the biofilm and the crystal regions.

##### 3.1.3.3 Biofilm-coated diatom units with protrusions (BCDU with protrusions)

**Fig. 5:**
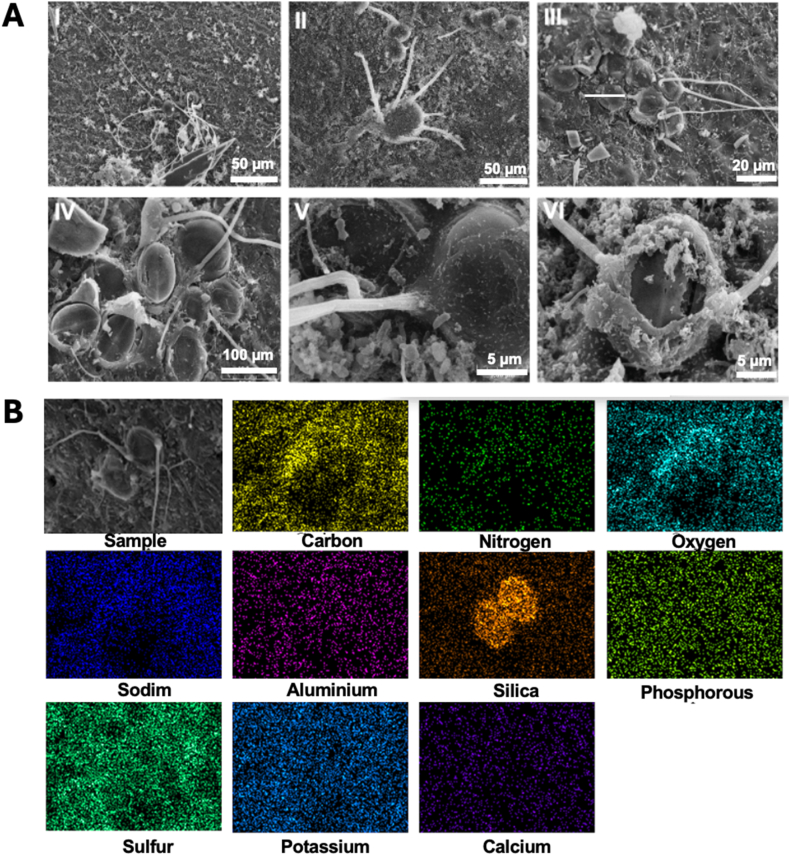
(**A**) **SEM images from the BCDU with protrusions from different samples of holopelagic *Sargassum* varieties**, *S. natans* var. *natans* (SNN) and *Sargassum fluitans* var. *fluitans* (SFF)*,* using different magnifications: I. SNN-fronds (500x), II. SFF-fronds (500x), III. SSF-fronds (1000x), IV. SFF-fronds (2500x), V. SNN-fronds (5000x), VI. SFF-vesicles (5000x). (**B**) **Elemental composition mapping of the BCDU with protrusions**.

SEM observations revealed the presence of structures resembling a shell with a diatom inside and with two to six protrusions or projections, depending on the diatom size (2 projections if structure length <20 μm and 6 projections if structure length >50 μm) (Fig. 5A & Fig. S7). These structures were absent on SNW, but were observed on the fronds, axes, and floating vesicles of SFF at both locations, and on nearshore fronds and floating vesicles of SNN. Further observations showed that when these “shells” were broken, most BCDU with protrusions contained a diatom identified as *Cocconeis* sp. (Fig. 5A–IV & VI), except for one unusually large - at least 10 times larger than the others containing *Cocconeis* sp. (Fig. 5A–II is at the same scale as Fig. 5A–I)- which contained an unidentified diatom. Elemental composition mapping confirmed that the shell structures were composed of Si (Fig. 5B), thereby verifying the presence of diatoms within these enclosed structures.

**Fig. 6:**
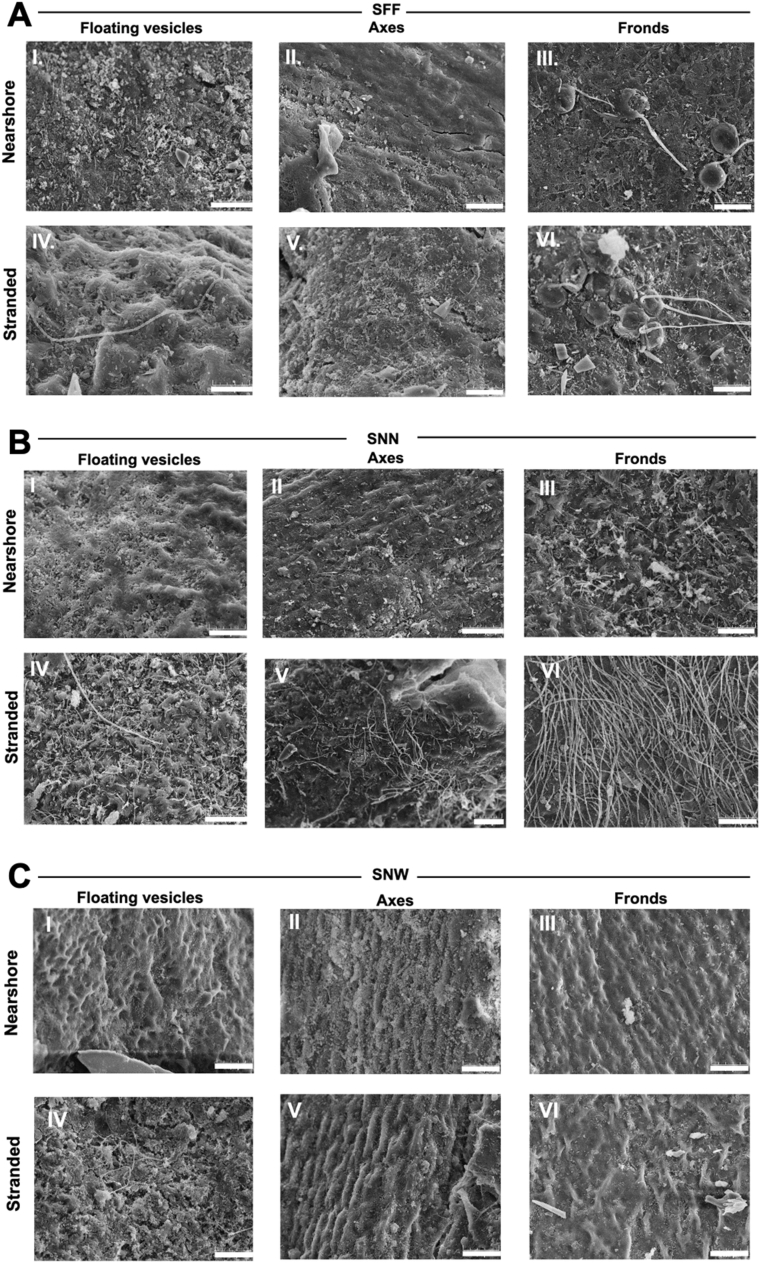
**SEM images of holopelagic *Sargassum*.** (A) *S. fluitans* var. *fluitans* (SFF), (B) *S. natans* var. *natans* (SNN), and (C) *S. natans* var. *wegei* (SNW). For each taxon, SEM images show floating vesicles, fronds, and axes under nearshore (I–III) and stranded (IV–VI) conditions: floating vesicles (I, IV), fronds (II, V), and axes (III, VI). Bars = 20 μm (1000x magnification).

### 3.2 Quantitative analyses

The quantitative analyses were designed to address the three hypotheses outlined above. Therefore, Section 3.2.1 focuses on intra-species differences at both locations, specifically, we examined the variability of biofilm characteristics among the different thallus parts (axes, fronds, and floating vesicles) (Fig. 1). Section 3.2.2 compares the three taxa across both locations (Fig. 6).Finally, Section 3.2.3 examines the effects of stranding on each species and identifies whether specific thallus parts are particularly affected (Fig. 6).

#### *3.2.1* *Variation in biofilm composition and development among thallus parts within* Sargassum *taxa*

SNW showed no significant differences in biofilm characteristics among thallus parts at either location (KW, *p* > 0.05 for all variables), indicating a stable biofilm structure irrespective of thallus part or environmental context. In contrast, SNN displayed the highest heterogeneity among thallus parts in biofilm characteristics. Stranding increased biofilm characteristics variability among thallus parts in both SNN and SFF.

**Fig. 7:**
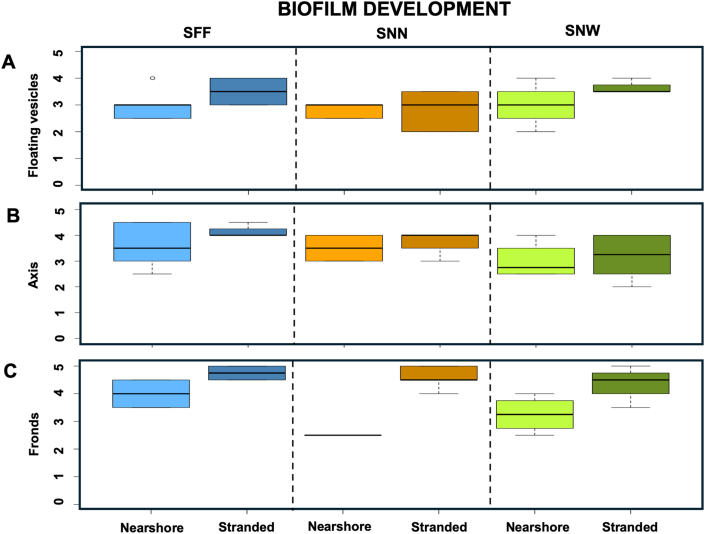
**Boxplots illustrating the biofilm classification across the three *Sargassum* taxa from nearshore and stranded specimens.** Panels depict the biofilm classification for: (**A**) floating vesicles, (**B**) axis, and (**C**) fronds (n = 4). The boxes span the interquartile range, from the 25th to the 75th percentiles of the data for each treatment, with the central horizontal line indicating the median. Whiskers extend to the 95% confidence intervals. *Sargassum fluitans* var. *fluitans* SFF, *S. natans* var. *natans* SNN, and *S. natans* var. *wingei* SNW.

**Fig. 8:**
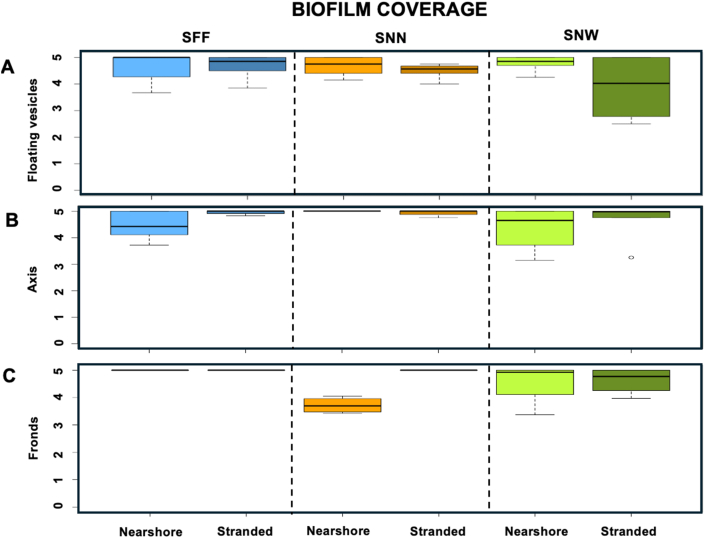
**Boxplots illustrating the percentage of biofilm coverage (%)** of the three *Sargassum* varieties from nearshore and stranded specimens. Panels depict the percentage coverage for: (**A**) floating vesicles, (**B**) axis, and (**C**) fronds (n = 4). The boxes span the interquartile range, from the 25th to the 75th percentiles of the data for each treatment, with the central horizontal line indicating the median. Whiskers extend to the 95% confidence intervals. *Sargassum fluitans* var. *fluitans* SFF, *S. natans* var. *natans* SNN, and *S. natans* var. *wingei* SNW.

**Fig. 9:**
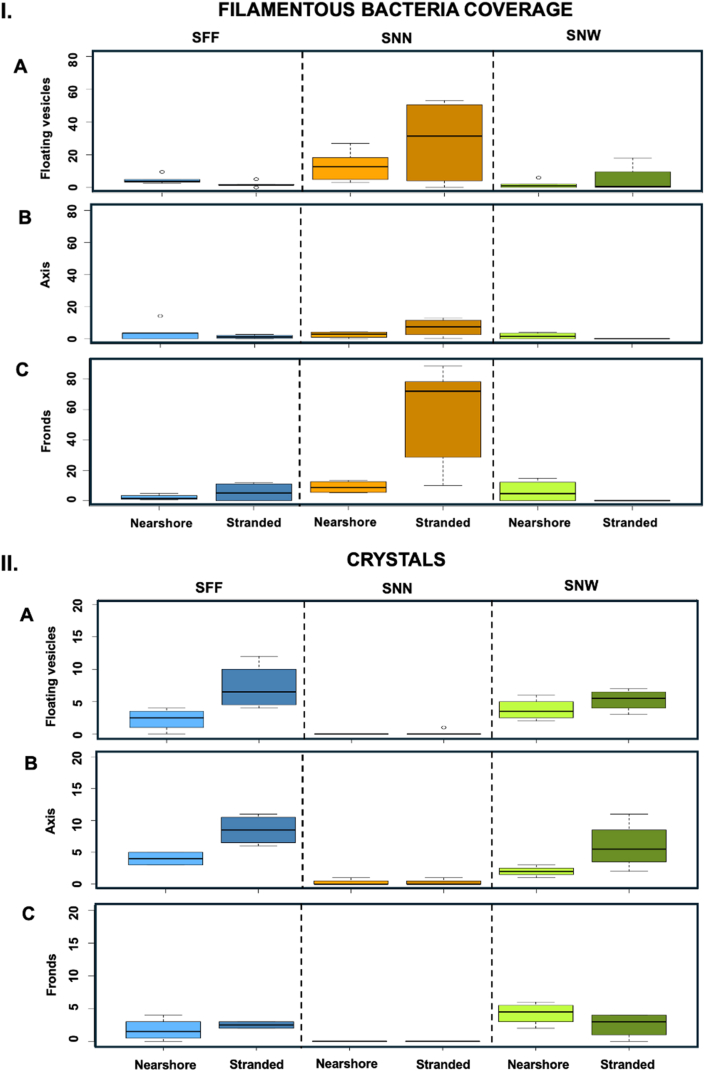
**I. Boxplots illustrating the percentage of superficial filamentous bacteria coverage (SFBC) (%) of the three *Sargassum* taxa from nearshore and stranded specimens.** Panels depict the percentage coverage for: (**A**) floating vesicles, (**B**) axis, and (**C**) fronds (n = 4). **II. Boxplots illustrating the density of crystals across the three *Sargassum* taxa from nearshore and stranded specimens.** Panels depict the number of crystals per surface area of 0.2 mm^2^ for: (**A**) floating vesicles, (**B**) axis, and (**C**) fronds (n = 4). The boxes span the interquartile range, from the 25th to the 75th percentiles of the data for each treatment, with the central horizontal line indicating the median. Whiskers extend to the 95% confidence intervals. Points beyond the whiskers represent outliers. *Sargassum fluitans* var. *fluitans* SFF, *S. natans* var. *natans* SNN, and *S. natans* var. *wingei* SNW.

Nearshore SNN exhibited pronounced spatial heterogeneity in biofilm characteristics. SNN-fronds showed significantly lower biofilm development and coverage than SNN-axes (KW, *p* < 0.05; Dunn's test, *p* < 0.05) (Fig. 7, Fig. 8), while simultaneously supporting higher bacterial densities than SNN-peduncles (*p* < 0.05). Although SFBC differed among thallus parts overall (KW, *p* < 0.05), no significant pairwise contrasts were detected (Dunn's test, *p* > 0.05), likely reflecting high variability among replicates (Fig. 9-I). Nevertheless, median SFBC was greater on SNN-fronds and SNN-vesicles than on SNN-axes (Fig. 9-I), indicating an inverse relationship between SFBC and biofilm development across SNN thallus parts.

When stranded, biofilm coverage was lowest on SNN-vesicles and higher on SNN-axes and SNN-fronds (KW, *p* < 0.01), with SNN-fronds showing greater biofilm development than SNN-vesicles (Dunn's test, *p* < 0.05). Similarly, SFF-fronds exhibited significantly higher biofilm development than SFF-vesicles (Dunn's test, *p* < 0.05), but no difference in SFBC or bacterial density were observed (KW, *p* > 0.05). SFF was also the only species to display significant variation in crystal abundance among thallus parts under stranded conditions (Kruskal–Wallis, *p* < 0.05), with SFF-axes hosting more crystals than SFF-fronds (Dunn's test, *p* < 0.05) (Fig. 9-II).

**Fig. 10:**
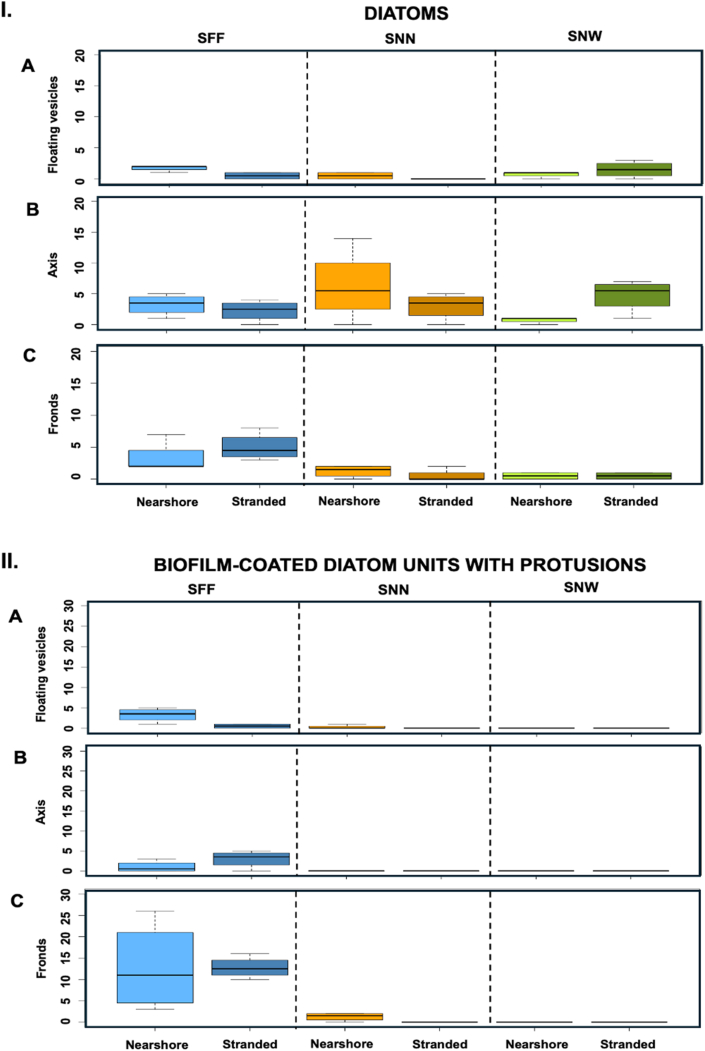
I. Boxplots illustrating diatom density across the three *Sargassum* taxa from nearshore and stranded **specimens**. Panels depict the number of diatoms per surface area of 0.2 mm^2^ for: (**A**) floating vesicles, (**B**) axis, and (**C**) fronds (n = 4). **II. Boxplots illustrating biofilm-coated diatom units with protrusions (BCDU with protrusions) density** across the three *Sargassum* taxa from nearshore and stranded specimens. Panels depict the number of BCDU with protrusions per surface area of 0.2 mm^2^ for: (**A**) floating vesicles, (**B**) axis, and (**C**) fronds (n = 4). The boxes span the interquartile range, from the 25th to the 75th percentiles of the data for each treatment, with the central horizontal line indicating the median. Whiskers extend to the 95% confidence intervals. *Sargassum fluitans* var. *fluitans* SFF, *S. natans* var. *natans* SNN, and *S. natans* var. *wingei* SNW.

Concerning the presence of diatoms and BCDU with protrusions, no consistent pattern was observed (Fig. 10). Nearshore, no taxon showed significant differences in diatom density among thallus parts (KW, *p* > 0.05), whereas BCDU with protrusions were more abundant on SFF axes than on SFF fronds (Kruskal–Wallis, *p* < 0.05; Dunn's test, *p* < 0.05).When stranded, SFF was the only taxon in which BCDU were observed and the only one to exhibit significant intra-thallus variation in diatom abundance and BCDU with protrusions (KW, *p* < 0.05). In this taxon, fronds displayed significantly higher abundances of both parameters than floating vesicles (KW, *p* < 0.05; Dunn's test, *p* < 0.05).

#### 3.2.2 Comparison in biofilm characteristics among taxa at each location

Comparisons among taxa revealed location-dependent responses (Table 1). Nearshore conditions highlighted taxon-specific biofilm architectures, whereas stranding reduced overall biofilm differences while enhancing contrasts in SFBC and crystal presence (Fig. 9). Overall biofilm coverage and bacterial density on fronds and peduncles of floating vesicles did not differ significantly among taxa for corresponding thallus parts in either nearshore or stranded samples (KW, *p* > 0.05), suggesting the same pattern of colonization. Nevertheless, differences in SFBC and biofilm development were apparent, with a consistent tendency toward higher SFBC in SNN. In nearshore samples, SFBC was higher on SNN-vesicles (12.6 ± 11.6%) than SNW-vesicles (1.0 ± 1.5%) (KW, *p* < 0.05) (Fig. 9-I). Fronds biofilm development was higher in SFF-fronds (4.0 ± 0.7%) than SNN-fronds (2.5 ± 1.0%) (KW, *p* < 0.05; Dunn's test, *p* < 0.05) (Fig. 7).

**Table 1:**
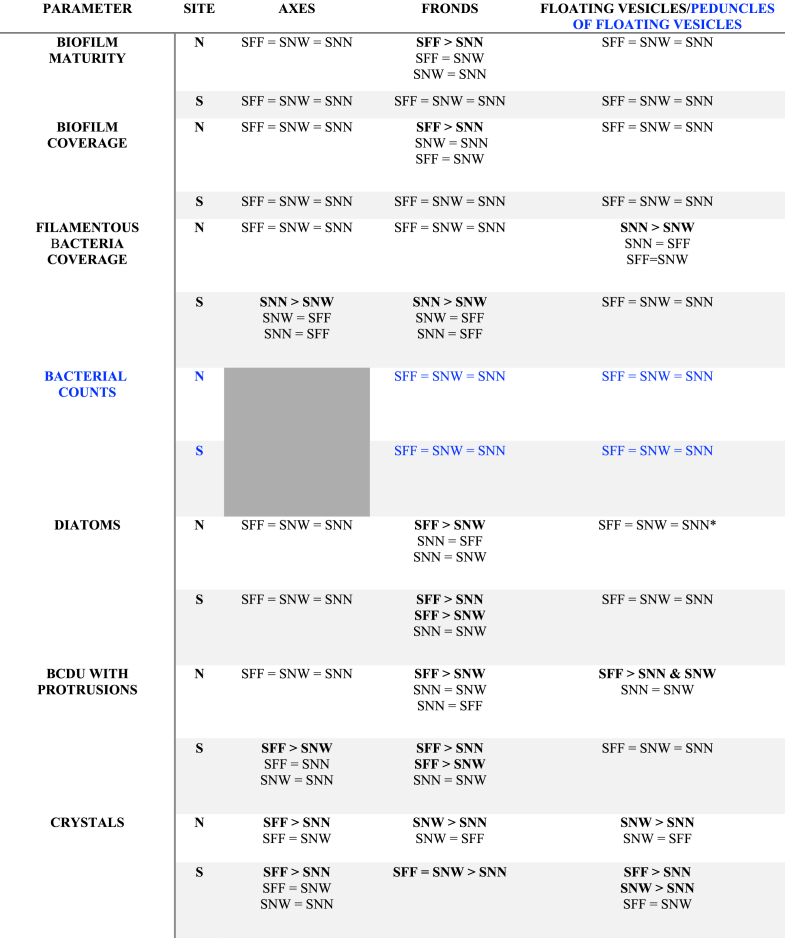
Summary of significant differences in biofilm characteristics among taxa for each thallus part. Depending on the line, data refer to either floating vesicles (black) or peduncles of floating vesicles (blue). Bacterial counts were conducted on fronds and peduncles of floating vesicles only (results presented in blue). Symbols: – = site effect not tested; N = nearshore; S = stranded; ∗ = significant Kruskal–Wallis test with non-significant post hoc comparisons.

Stranding eliminated differences in biofilm development and coverage among taxa (Fig. 7, Fig. 8). Despite axes did not show changes on biofilm development after stranding, SFBC on SNN-axes (7.5 ± 6.0%) and SNN-fronds (72.0 ± 24.0%) was higher than on SNW-axes and SNW-fronds (no visible coverage on all samples), respectively.

Crystal abundance showed distinct and often opposite patterns to SFBC. Overall, SNN consistently displayed lower crystal abundance than the other taxa (Fig. 9-II). When stranded, crystal abundance on SNW and SFF axes was significantly higher than on SNN axes (KW, *p* < 0.05). Similarly, stranded SNW fronds hosted significantly more crystals than SNN fronds (KW, *p* < 0.05). On floating vesicles under nearshore conditions, SFF vesicles exhibited higher crystal abundance than SNN vesicles (KW, *p* < 0.05). Together, these patterns support a negative association between SFBC dominance and crystal presence.

#### 3.2.3 Which thallus components are first impacted by stranding, and how do biofilm characteristics within these parts differ between nearshore and stranded specimens?

Location effects differed markedly between biofilm structure and its components, with axes consistently emerging as the least affected thallus parts across taxa. Biofilm development and coverage were influenced by location only in SNN, where stranded fronds exhibited significantly higher values than nearshore fronds (Fig. 7, Fig. 8) (KW, *p* < 0.05).

Stranding strongly increased bacterial colonization across taxa (KW, *p* < 0.001). On fronds, bacterial densities were nearly threefold higher in stranded than in nearshore individuals of both SNN and SNW, ranging from 2.13 × 10^4^ to 5.85 × 10^4^ cells mm^−2^ in SNN-fronds and from 1.34 × 10^4^ to 3.87 × 10^4^ cells mm^−2^ in SNW-fronds. However, SFF-fronds were unaffected (median: 2.46 × 10^4^ cells mm^−2^). In contrast, bacterial densities of peduncle from floating vesicles were significantly affected by stranding only in SFF, where stranded peduncles of floating vesicles exhibited nearly fivefold higher densities than nearshore ones (median: 4.04 × 10^4^ vs. 8.03 × 10^3^ cells mm^−2^; KW, *p* < 0.01).

SFBC did not differ significantly between locations (KW, *p* > 0.05), likely due to high variability among replicates; nevertheless, median coverage in SNN-fronds and SNN-vesicles was approximately twice higher in stranded than in nearshore samples (Fig. 9-I).

BCDU with protrusions were more abundant on SFF-vesicles in nearshore than in stranded samples (Fig. 10-II) (KW, *p* < 0.05). Conversely, diatom presence increased on SNW-axes and SFF-vesicles under stranded conditions (Fig. 10-I) (KW, *p* < 0.05), whereas crystal abundance also increased significantly in stranded SFF-fronds and SFF-vesicles (Fig. 9-II) (KW, *p* < 0.05).

#### 3.2.4 Relationship between the presence of superficial filamentous bacteria coverage (SFBC) and crystal presence

Kendall's rank correlation was used to assess monotonic associations between variables without assuming linearity or normality. When analyzed all samples combined, a significant negative correlation was observed between SFBC and crystal abundance (Kendall's τ = −0.37, *p* < 0.001), indicating a moderate inverse relationship in which higher crystal abundance is associated with lower SFBC. When stranded and nearshore samples were analyzed separately, the correlation remained significant only for stranded samples (Kendall's τ = −0.35, *p* < 0.01), whereas it was not significant for nearshore samples (Kendall's τ = −0.22, *p* > 0.05). The lack of significance in nearshore samples is likely due to the low abundance of SFBC, irrespective of crystal presence. Overall, this pattern suggests a potential biological interaction in which crystal occurrence may limit SFBC in stranded samples. For plotting the data, values were transformed using log_10_(x + 1) to reduce distributional skewness and minimize the influence of extreme values. The relationship does not appear to be strongly linear, as indicated by the low coefficient of determination obtained from the regression analysis (Fig. S8). This pattern may be influenced by contrasting distributions among sample types, with crystals being rare in SNN samples irrespective of SFBC, and SFBC being scarce in SNW samples regardless of crystal abundance.

## 4 Discussion

### 4.1 Elemental composition of holopelagic *Sargassum* biofilms

Biofilms are primarily composed of extracellular polymeric substances (EPS), including proteins, nucleic acids, and polysaccharides [8]. While the elemental composition of whole macroalgal tissues has been studied using ground material [38], the composition of macroalgal biofilms remains largely unexplored. Comparisons with marine biofouling biofilms and single-cell freshwater bacterioplankton reveal both similarities and differences in the elements detected [39,40]. Notably, C and O are reported exclusively in biofilms, likely reflecting the high abundance of EPS. Among macro-minerals, only Ca was consistently observed across all three studies. Elements uniquely detected in our study include N, Mg, P, S, K, Al, and As. In contrast, trace metals such as Fe, Cu, Pb, and Ni were reported in the previous studies but were not detected in our samples.

The presence of C and O aligns with their incorporation in polysaccharides, while sulfur may derive from structural biomolecules such as proteins and sulfated polysaccharides (e.g. fucoidans), or from H_2_S during algal degradation following stranding [41]. Phosphorus likely reflects its presence in nucleic acids and phosphorylated proteins [19]. Silica, commonly linked to diatoms [42], was only observed in diatoms and BCDU with protrusions in our study.

We also detected crystals composed of Ca, P, and O, consistent with calcium phosphate, representing the first report of such crystals in holopelagic *Sargassum* biofilms (Fig. 4). Previous studies reported CaCO_3_ in whole tissue samples [43]. In our study, only Ca–P–O crystals were observed, though Ca was also present in non-crystalline form in other biofilm areas. Additionally arsenic and aluminium were detected across all three taxa, suggesting biofilms may participate in early heavy metal accumulation. Brown seaweeds, including holopelagic *Sargassum*, are known to accumulate metals such as arsenic via cell wall interactions (Gobert et al., [44]; Alleyne et al., 2023; McGillicuddy et al., 2023). Our observations suggest that biofilms, potentially mediated by microbial taxa such as *Pseudomonas* (bacteria) and *Aspergillus* (fungi), both identified in holopelagic *Sargassum*, may play a role in arsenic cycling and transformation [[45], [46], [47], [48], [49]]. This highlights the role of biofilms in shaping elemental distribution on macroalgal surfaces and adds a new perspective to *Sargassum*–microbe interactions and biogeochemical dynamics.

### 4.2 Ca–P–O crystals as part of holopelagic *Sargassum* biofilms

The formation of calcium-containing crystals in holopelagic *Sargassum* biofilms appears to be closely linked to localized pH fluctuations within the seaweed diffusive boundary layer (DBL), driven by macroalgal and microbial processes. Photosynthetic activity in holopelagic *Sargassum* during daylight raises pH at the DBL, creating favorable conditions for Ca–P–O crystal formation. Microbial processes, particularly denitrification, can further increase local pH (Arvin and Kristensen, [50]; Kolodkin-Gal et al., 2023). The higher abundance of Ca–P–O crystals in stranded SFF coincides with the increase of denitrifying bacteria as *Sargassum* holobionts approach the coast (Léger-Pigout et al., [51]). Night-time respiration, which increases CO_2_ concentrations within biofilms at the DBL, may also contribute to crystal nucleation and growth [52]. Collectively, these processes, together with the heterogeneous distribution of microbial communities and biofilm development, suggest that the interplay between algal photosynthesis and microbial activity generates dynamic physico-chemical microenvironments, conductive to Ca–P–O crystal formation on *Sargassum* surfaces. Moreover, the EPS matrices may further facilitate localized nucleation and precipitation of crystals [53].

Ca crystals have been associated with enhanced biofilm stability by reinforcing structural integrity, reducing cell dispersal, and promoting the development of thicker, more wrinkled biofilm matrices, that improve resistance to shear stress [54,52,55]. In our study, although Ca–P–O crystals were clearly identified, Ca was also detected in non-crystalline forms within biofilm areas lacking visible crystals (Fig. 5B), suggesting multiple modes of calcium incorporation within the biofilm. Ca–P–O crystal formation may be modulated by the macroalgal host's phosphorus metabolism, as well as by associated microbial communities involved in phosphorus remineralization, including Pseudomonadota, Bacteroidota, Cyanobacteriota, and Planctomycetota [56]. In addition, bacterial genera capable of precipitating Ca–P–O crystals, such as *Pseudomonas* [55], have been reported as abundant members of holopelagic *Sargassum* biofilms (Theirlynck et al., 2023), supporting a potential microbial contribution to crystal formation.

Surface properties may also play a role in biofilm formation and composition. In particular, surface texture and roughness, known to influence the creation of microhabitats [57], -could modulate both biofilm development and crystal precipitation. In this context, SNN coincided with higher SFBC, lower biofilm stability, and lower occurrence of Ca–P–O crystals, suggesting a potential link between crystal formation and biofilm structural integrity. Overall, differences in microbial community composition and distribution among SNN, SNW, and SFF are likely related to variation in Ca–P–O crystal formation and biofilm resilience, with the relationship potentially operating in either direction.

### 4.3 Diatoms and BCDU with protrusions

Assessing the role of diatoms on macroalgal surface remains complex, as their abundance is influenced by multiple factors, including host morphology, environmental conditions, and tissue state [[58], [59], [60]]. Since all taxa were sampled under identical environmental conditions, these differences likely reflect taxon-specific traits. Highly branched seaweeds with articulated thalli tend to favor diatom colonization [58], which is consistent with our observations of higher diatom abundance on SFF. Diatoms were not dominant in any thallus part, with adnate forms prevailing, in contrast to reports of erect and motile diatoms on Icelandic seaweeds [58]. Our findings instead align with studies showing preferential colonization of damaged tissues and microstructural features without negative effects on host health [61]. Consistently, stranding was associated with increased diatom abundance in SFF and SNW, with differences among thallus parts emerging only under stranded conditions, a pattern not observed in SNN.

Diatom-bacteria interactions are known to involve symbiotic processes that shape microbial communities [62,63]. In line with this, taxa exhibiting increased diatom abundance upon stranding (SFF and SNW) showed reduced SFBC and greater biofilm stability, suggesting a link between diatoms, bacterial partners and overall biofilm composition (Fig. 6, Fig. 9). Similarly, studies on holopelagic *Sargassum* biofilms report comparable microbial families across taxa, but with differences in relative abundances and diversity [64]. Our results indicate that these differences extend to biofilm structural traits, potentially including EPS components. Together, these patterns highlight the need to better understand chemical communication between microbial partners and their macroalgal hosts within biofilm communities.

The structures observed covering diatoms (BCDU with protrusions) remain unresolved but may be associated with EPS, as they were not visible under confocal microscopy (Fig. S7A). Previous SEM observations of *Sargassum* have reported diatoms such as *Cocconeis* spp., in close proximity to the host's cryptostome [65], suggesting that these features may reflect specific host-microbiome interactions. The marked variation in BCDU with protrusions among taxa, that are abundant in SFF and absent in SNW, highlights potential interspecific differences. Elucidating their formation and function could provide further insight into the ecological and structural dynamics of *Sargassum* biofilms.

### 4.4 Heterogeneity of seaweed surfaces, biofilms, and associated structures

The heterogeneity observed in macroalgal biofilms likely arises from multiple interacting factors, including macroalgal biochemical composition and surface-released metabolites [[66], [67], [68], [69]], as well as the physico-chemical environment at the DBL [70,71] and surface topography. In the present study, both qualitative observations of surface texture and 2D-based estimations of surface roughness indicate variation in surface topography across thallus parts and among *Sargassum* taxa. Such variability may have important ecological implications for microbial colonization. While rougher surfaces are known to enhance microbial attachment on inert surfaces [[72], [73], [74]], their role in biological systems, including macroalgae, remains largely unexplored, as most studies have focused on chemical drivers of microbial selection (Egan et al., 2013; Coelho-Souza et al., [75]; Abdul Malik et al., [76]; Paix et al., 2020). In addition to chemical drivers, the external environment, including seawater hydrodynamics, as well as the interactive physiological responses of the host and its microbiome across different surface topographies, modulate the physicochemical conditions within the DBL. These factors can favor or inhibit specific microbial assemblages; therefore, a holistic approach is required to fully understand biofilm heterogeneity. Regardless of the specific contribution of these factors, we observed marked heterogeneity in the abundance of diatoms, BCDU with protrusions, and crystals across the three *Sargassum* taxa and their respective thallus parts. SFF supported the highest densities of all three components, suggesting taxon-specific patterns. In contrast, SFBC were predominantly observed on the floating vesicles and fronds of stranded SNN. Moreover, the negative correlation between crystals presence and SFBC, suggests potential interactions, either direct, where crystals may inhibit filamentous bacterial development, or or indirect, mediated by the physicochemical environment or microbial assemblages that favor one over the other. Nevertheless, additional drivers, such as biochemical cues and environmental exposure, are also likely to contribute [66]. Overall, the association of crystals with more complex and stable biofilms [55], may partly explain the greater biofilm stability observed after stranding in SFF and SNW fronds compared to SNN fronds.

### 4.5 Shifts in biofilm and epiphytic composition across locations (stranded and nearshore) and seaweed taxa

Biofilms growing on living surfaces can modulate the exchange of signals, energy, and matter across the host interface [77], but their maturity and coverage are not straight forward indicators of host health, as their effects depend on community composition and environmental conditions [77]. Independent observations of stranded *Sargassum* showed no photosynthetic activity (Connan, data not shown), while lipid content analyses of samples collected at the same locations (nearshore and stranded) revealed increased saturated fatty acids (SFAs) and decreased polyunsaturated fatty acids (PUFAs) in stranded samples, consistent with progressive degradation [4].

In this context, our results highlight complex relationships between biofilm stability, thallus part, and taxon resilience. Biofilm development did not scale linearly with bacterial abundance, and increased bacterial colonization upon stranding was not always accompanied by enhanced biofilm maturity. Across taxa, axes consistently supported more stable biofilms, with no significant differences observed between stranded and nearshore samples. In contrast, fronds and floating vesicles were more prone to detachment and degradation. Overall, fronds—particularly those from SNN—represent the most vulnerable thallus components to degradation under stranded conditions. This is particularly significant because fronds are the primary sites of photosynthesis [78]; their deterioration leads to a collapse in the organism's fitness [79], resulting in overall decline. Similarly, the loss of floating vesicles causes the thallus to sink, ultimately resulting in death.

A habitat-driven degradation gradient was evident, with bacterial colonization increasing in holopelagic *Sargassum* fronds, regardless of the taxon, as rafts drifted toward the coast. Among taxa, SNN appeared most vulnerable, showing low biofilm development nearshore but a sharp increase in biofilm coverage and SFBC upon stranding. These patterns suggest that limited biofilm development in nearshore conditions may compromise structural integrity. In contrast, SNW and to a lesser extent SFF, maintained consistent biofilm coverage across both locations, with limited SFBC proliferation in SFF and none in SNW. This stability may be linked to the presence of Ca-P-O crystals, which are associated with increasing biofilm resistance [55]. Finally, the observation of bacteria with *Vibrio*-like morphology (Fig. S3) is consistent with metabarcoding data from samples collected at nearby locations [80].

## 5 Conclusions

Our sample size remains limited relative to the extensive spatial distribution of holopelagic *Sargassum* strandings. Nevertheless, we detected significant differences in biofilm characteristics both within species (among thallus parts) and among taxa, consistent with previous studies on seaweed microbiomes [81,82]. Comparing three holopelagic *Sargassum* taxa at nearshore and stranded sites revealed that biofilm traits under oceanic conditions influence each taxon's stability and response to stranding. Taxa with higher biofilm development, surface coverage, and structural components (e.g., Ca–P–O crystals) under nearshore conditions tended to maintain greater stability after stranding.

Intra-thallus comparisons revealed that fronds, the main photosynthetic organs, are particularly prone to degradation, driving the organism's overall decline. While overall biofilm maturity did not consistently change upon stranding, biofilm composition, and particularly mineral structures, appears to play a more decisive role in microbiome structure. Bacterial abundance generally increased across all taxa, but SFBC increased significantly only in SNN, the taxon with lower crystal content and potentially lower resilience, highlighting the importance of considering the full spectrum of biofilm constituents when evaluating holobiont health.

Finally, our study extends previous microscopic, molecular, and metabolomic findings [8,83,77,[81], [82], [84], [85], [86], [87]] by providing a three-dimensional perspective on intra-thallus and interspecies biofilm differences previously reported in the literature. We show that variation in macroalgal surface traits and microbiome components across taxa and thallus parts, interacting with environmental context, shapes biofilm three-dimensional structure and stability, with potential implications for host resilience.

## CRediT authorship contribution statement

**Zujaila Nohemy Qui-Minet:** Writing – review & editing, Writing – original draft, Visualization, Methodology, Investigation, Funding acquisition, Formal analysis, Conceptualization. **Christine Paillard:** Writing – review & editing, Validation, Methodology, Investigation, Formal analysis. **Valérie Michotey:** Writing – review & editing, Visualization, Investigation. **Solène Connan:** Writing – review & editing, Validation, Supervision, Methodology, Investigation, Conceptualization. **Philippe Elíes:** Visualization, Methodology, Investigation. **Valérie Stiger-Pouvreau:** Writing – review & editing, Validation, Supervision, Resources, Methodology, Investigation, Funding acquisition, Conceptualization.

## Declaration of competing interest

The authors declare that they have no known competing financial interests or personal relationships that could have appeared to influence the work reported in this paper.

## Data Availability

No data was used for the research described in the article.
