# Supplementary material for: Unraveling the hidden world: Variability and complexity of holopelagic Sargassum biofilms
Source: Biofilm. 2026 Apr 16;11:100362. doi: 10.1016/j.bioflm.2026.100362 (PMC13147391; doi:10.1016/j.bioflm.2026.100362)
Supplement: Multimedia component 1 [file mmc1.docx]

**Supplementary Material**

**for**

**Unraveling the Hidden World: Variability and Complexity of Holopelagic *Sargassum* Biofilms**

Zujaila Nohemy Qui-Minet^1*,^ Christine Paillard^1^, Solène Connan^1^, Philippe Elíes^1^, Valérie Stiger-Pouvreau^1^

*^1^ Univ Brest, CNRS, IRD, Ifremer, LEMAR, F-29280 Plouzane ́*

**Content**

Figure S1: SEM images of diatoms present in the superficial layer of the biofilm on holopelagic Sargassum taxa.

Figure S2. SEM images of *Sargassum natans* var. *natans* showing a soft, pendant lateral branch (~20 µm in width and >1 mm in length) arising from the main axis at different magnificaitons.

Figure S3. SEM images showing the presence of curved rod-shaped bacteria identified as *Vibrio* sp. within holopelagic *Sargassum* biofilms.

Figure S4: Energy-Dispersive X-ray Spectroscopy (EDS) representing the elements present within the 1 µm of the surface of holopelagic *Sargassum* species

Figure S5: Close-up of EDS spectrum of the holopelagic *Sargassum* samples. The Mg Kα (1.25 keV) and As Lα (1.28 keV) peaks overlap, indicated by the shaded region

Figure S6: SEM images of Ca–P–O crystals merged with biofilms and observed on different thallus parts of *Sargassum fluitans* var. *fluitans* (SFF) and *Sargassum natans* var. *wingei* (SNW). Arrows indicate areas where Ca–P–O crystals are mixed with the biofilm (A, B, C, E, F, G, H) or intertwined with biofilm components (D).

Figure S7: Biofilm-coated unit protrusions observed in SFF frond samples (indicated by arrows).

Figure S8: Correlation between log-transformed crystal abundance and log-transformed filamentous bacteria coverage in holopelagic *Sargassum (S. fluitans* var. *fluitans, S. natans v*ar. *natans and S. natans* var. *wingei)*.

Table S1: Parameters associated with surface roughness and surface smoothness measured at four orientations in the three taxa (SFF, SNN, SNW).

**Supplementary Material**


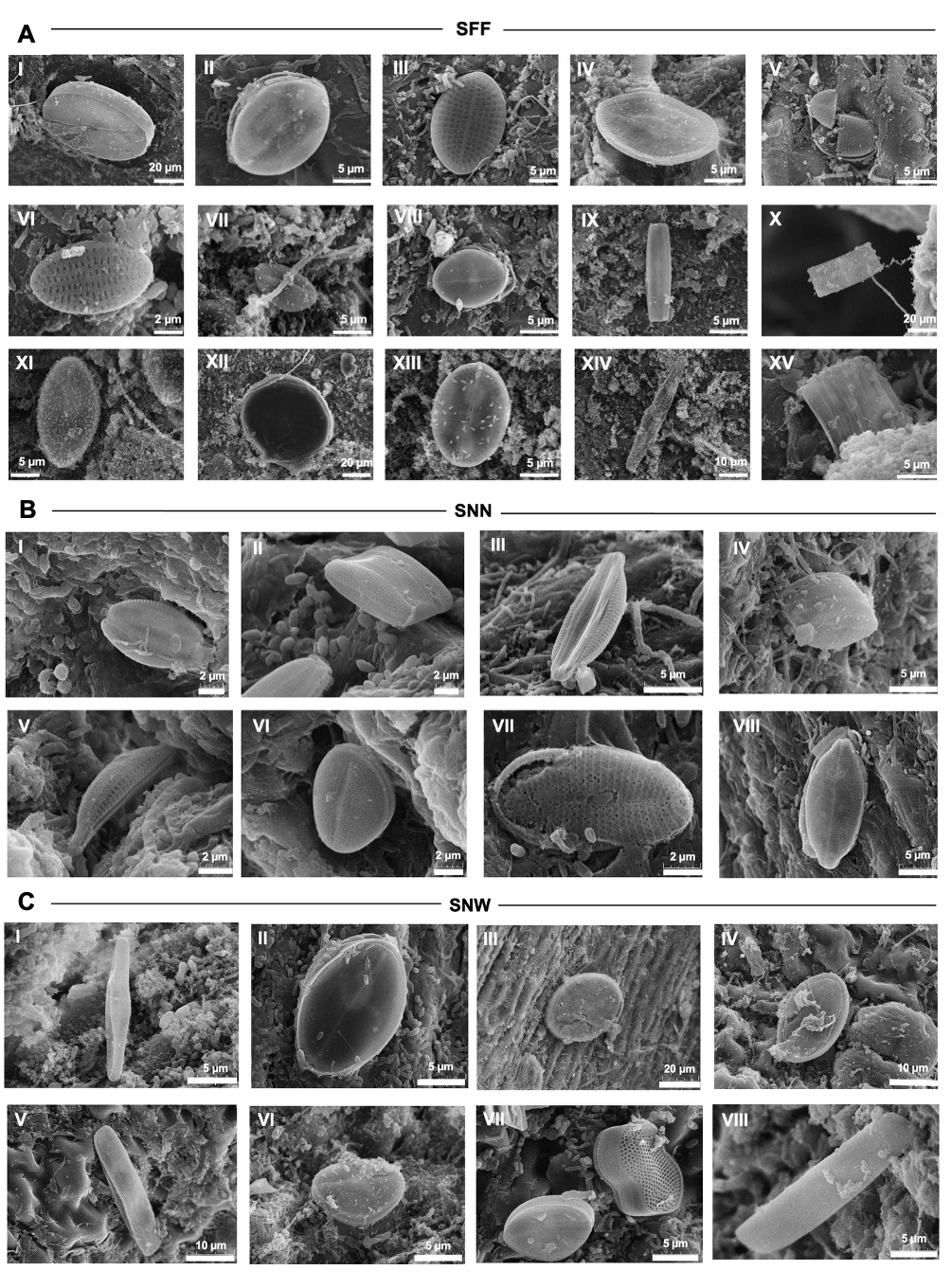


**Figure S1.** SEM images of diatoms present at the superficial biofilm layer of (A). *Sargassum fluitans* var. *fluitans*, (B) *Sargassum natans* var. *natans*, (C)*Sargassum natans* var. *wingei.*


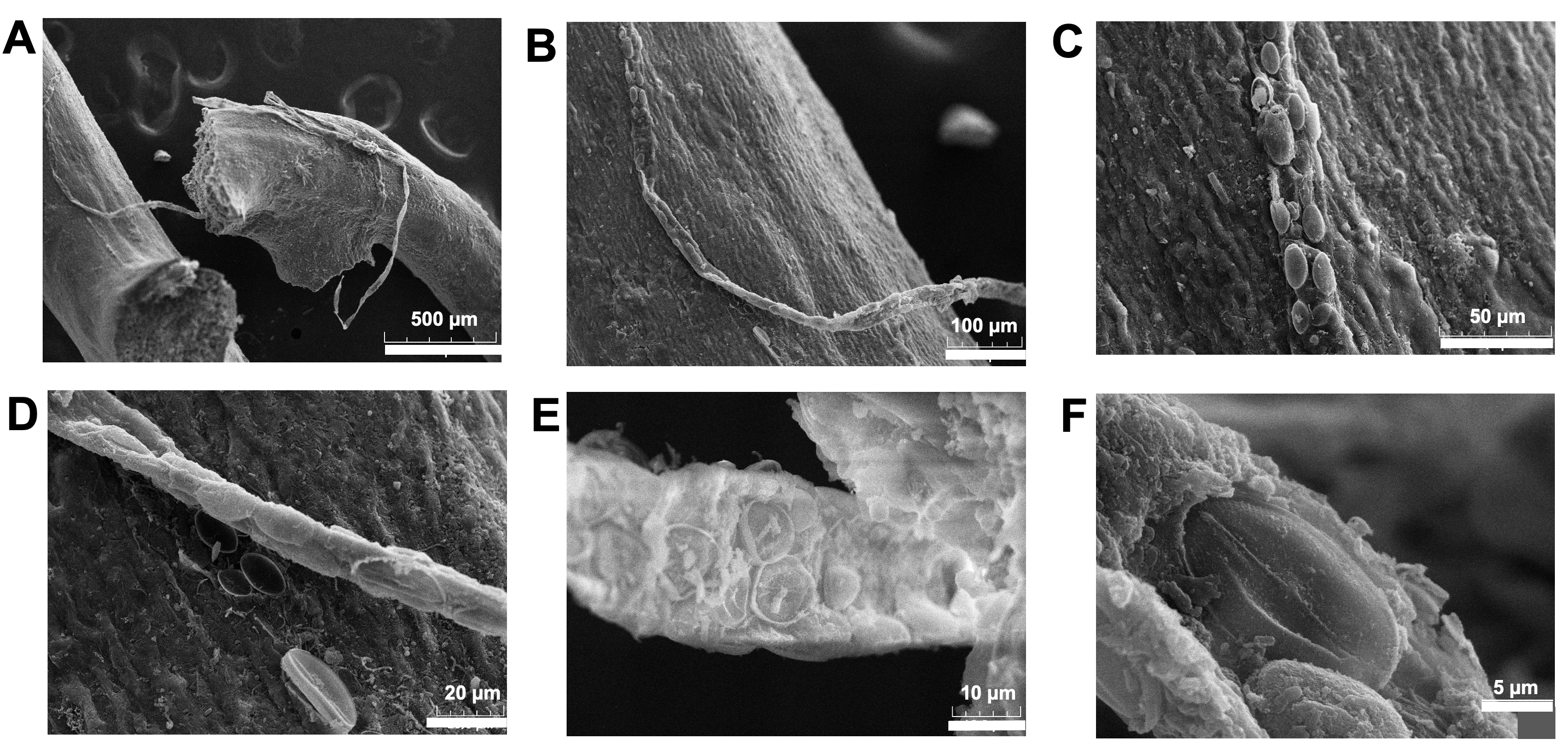


**Figure S2.** SEM images of *Sargassum natans* var. *natans* showing a soft, pendant lateral branch (~20 µm in width and >1 mm in length) arising from the main axis at increasing magnifications, revealing a dense and continuous diatom cover. Magnifications: (**A**) 30x  (**B**) 50x (**C**) 500x (**D**) 1000x (**E**) 2500x (**F**) 5000x.


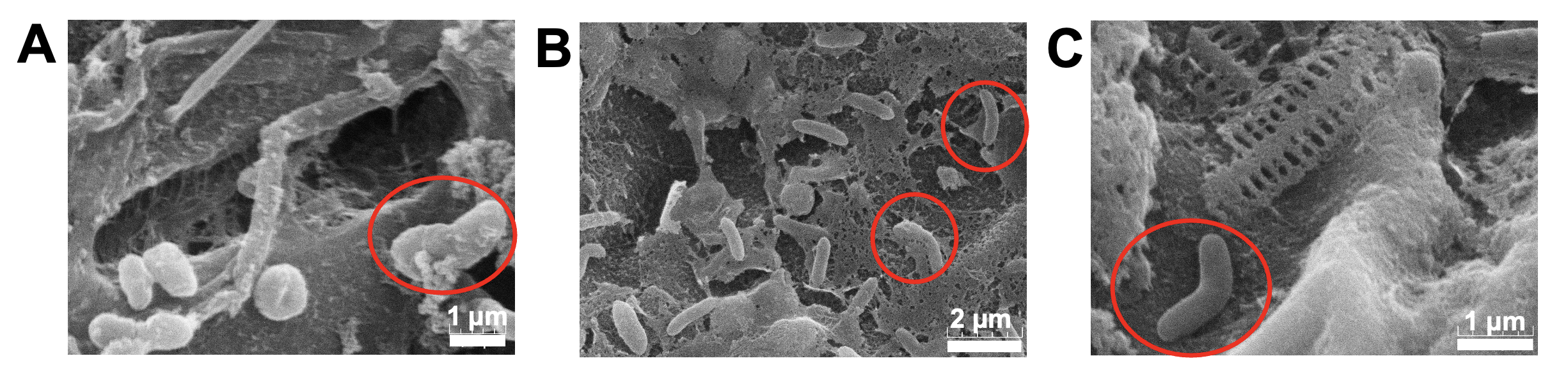


**Figure S3.** SEM images of biofilms associated with (**A**) *Sargassum fluitan*s var. *fluitans* (SFF), (**B**) *Sargassum natans* var. *natans* (SNN), and (**C**) *Sargassum natans* var. *wingei* (SNW), showing the presence of curved rod-shaped bacteria (circled in red), identified as *Vibrio* sp.

**
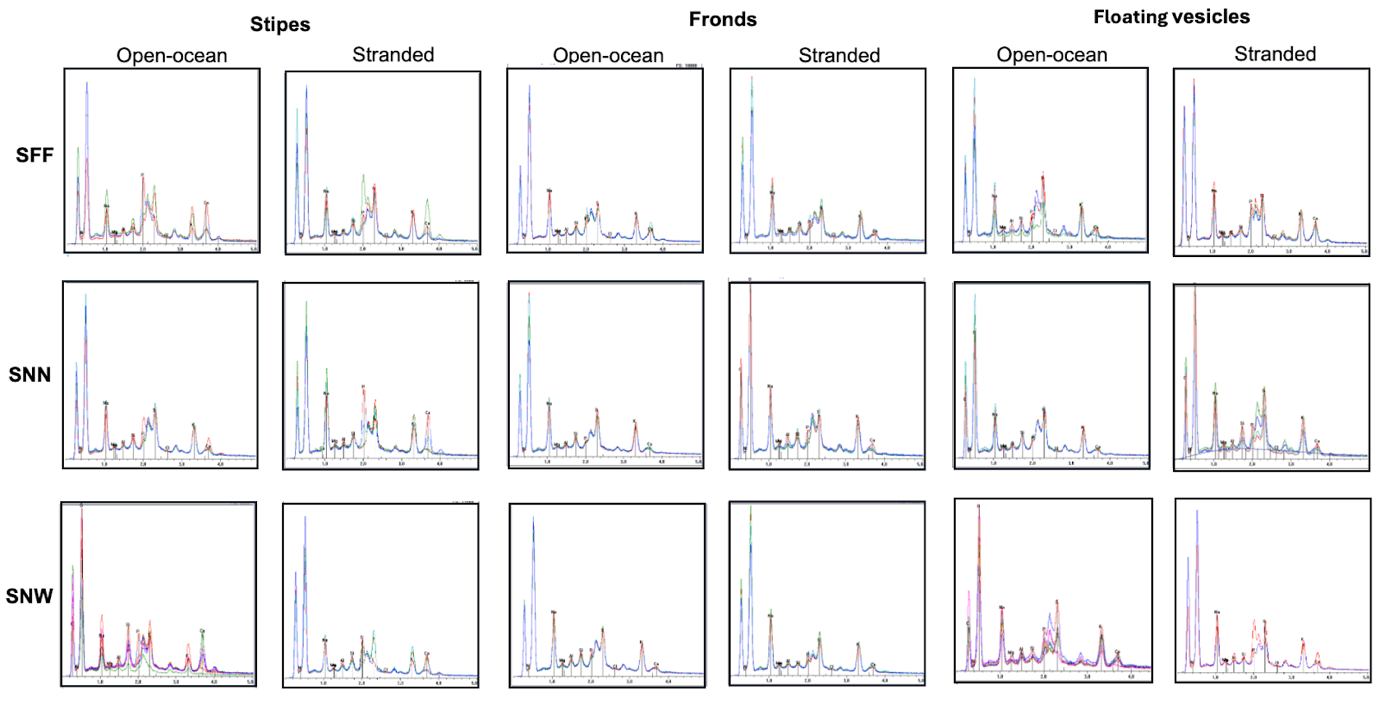
**

**Figure S4.** Energy-Dispersive X-ray Spectroscopy (EDS) representing the elements present within the 1 µm of the surface of holopelagic *Sargassum* species (each color represents a replica of the spectra, n=3)*.* Data is presented for nearshore and stranded samples from SFF, SNN and SNW axes, fronds and floating vesicles. X-axis: Energy (keV); Y-axis: X-ray counts.


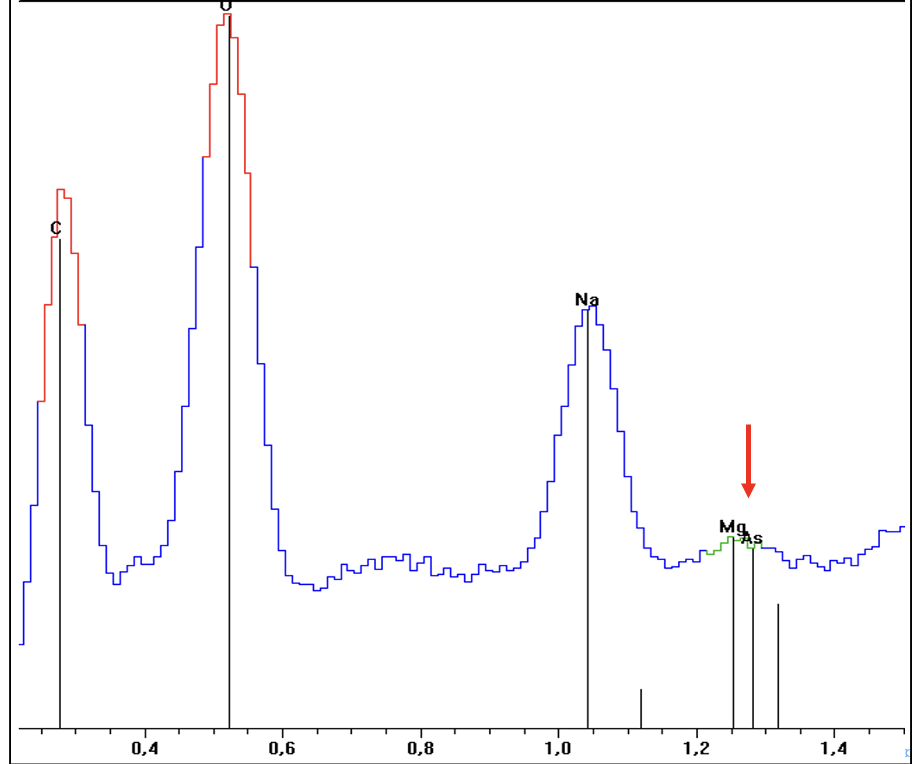


**Figure S5.** Close-up of EDS spectrum of the holopelagic Sargassum samples. The Mg Kα (1.25 keV) and As Lα (1.28 keV) peaks overlap, indicated by the  red arrow. X-axis: Energy (keV); Y-axis: X-ray counts.

**
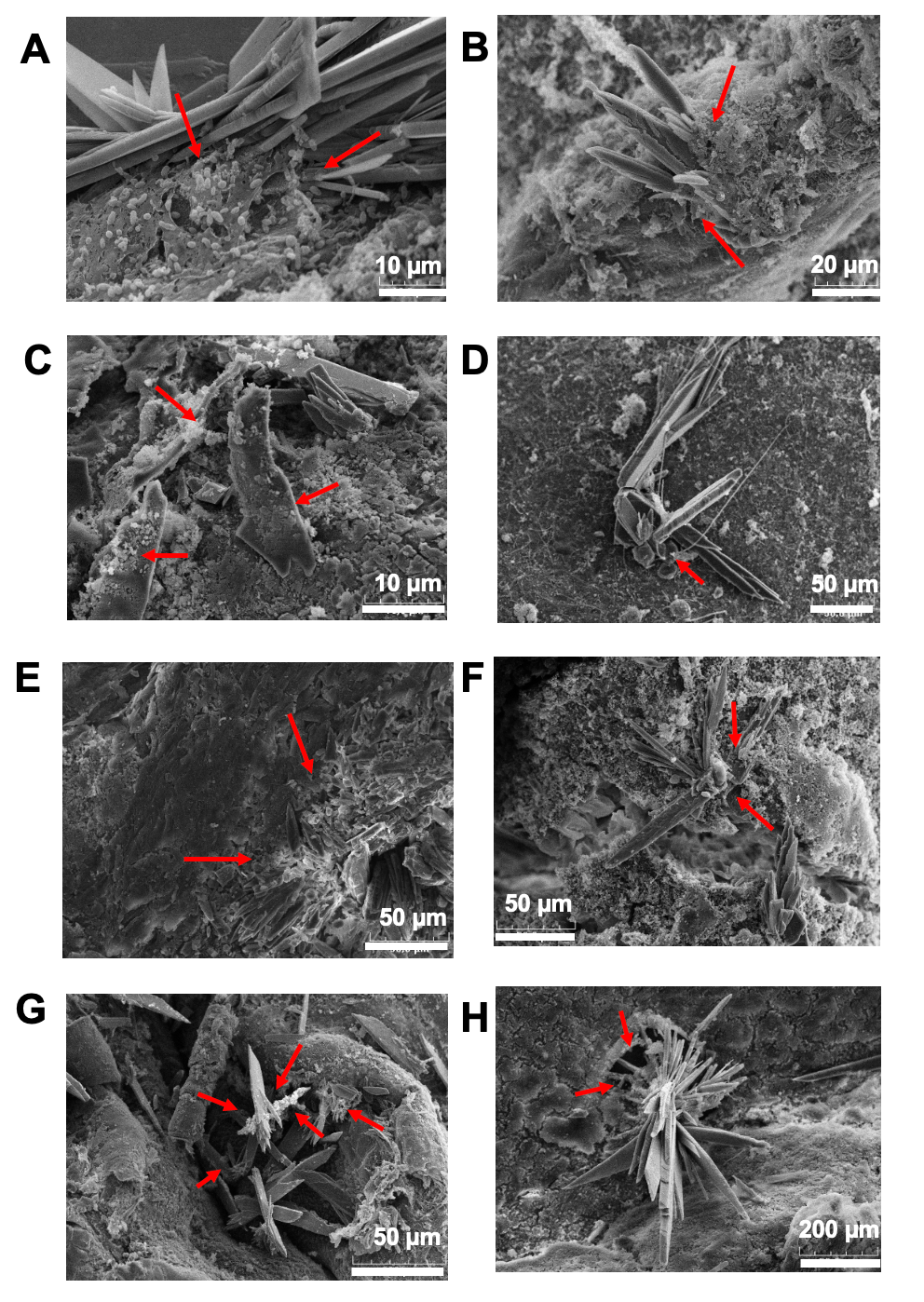
**

**Figure S6.** SEM images of Ca–P–O crystals observed on different thallus parts of *Sargassum fluitans* var. *fluitans* (SFF) and *Sargassum natans* var. *wingei* (SNW), at different magnifications. Images correspond to: (A, B) nearshore SFF axes (2500× and 1000×), (C, G) nearshore SNW floating vesicles (2000× and 500×), (D) nearshore SFF fronds (500×), (E) stranded SNW axis (500×), (F) stranded SFF axis (500×), and (H) nearshore SNW fronds (150×). Arrows indicate areas where Ca–P–O crystals are mixed with the biofilm (A, B, C, E, F, G, H) or intertwined with biofilm components (D).

**
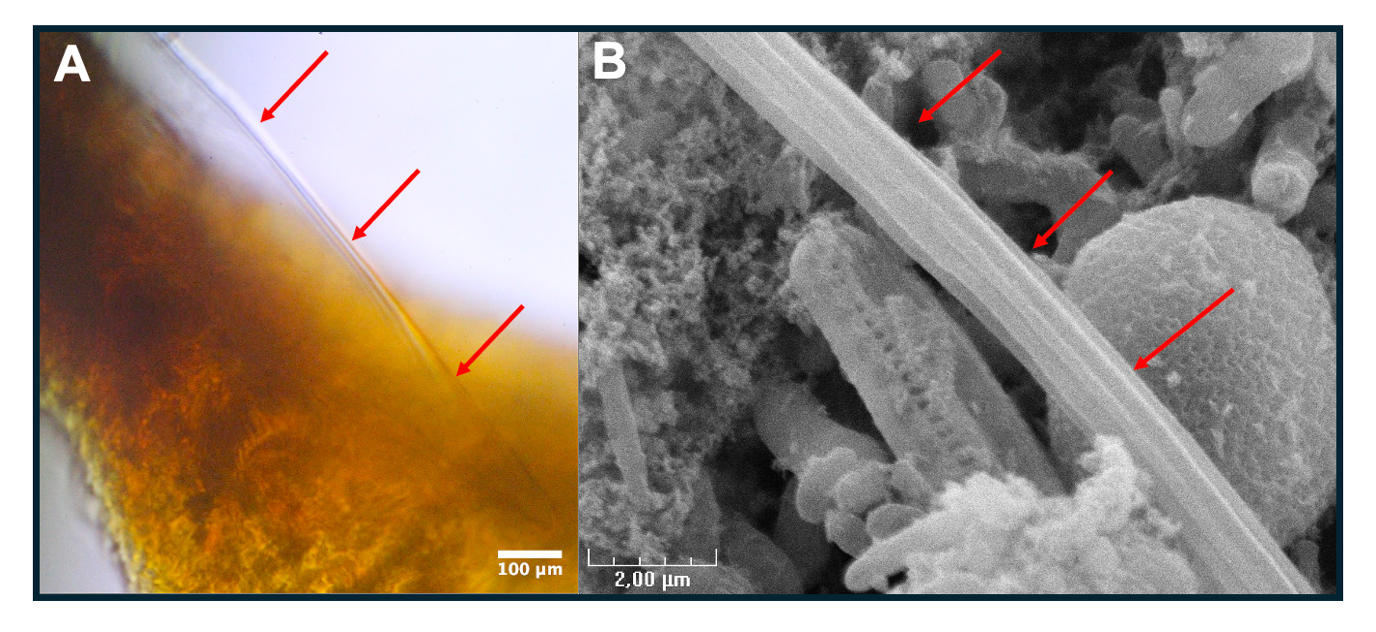
**

**Figure S7.** Biofilm-coated unit protrusions observed on *Sargassum fluitans* var. *fluitans* SFF frond samples (indicated by arrows). (A) Confocal microscopy image (10× magnification). (B) Scanning electron microscopy close-up (10,000× magnification) showing internal filaments within the protrusions.

**
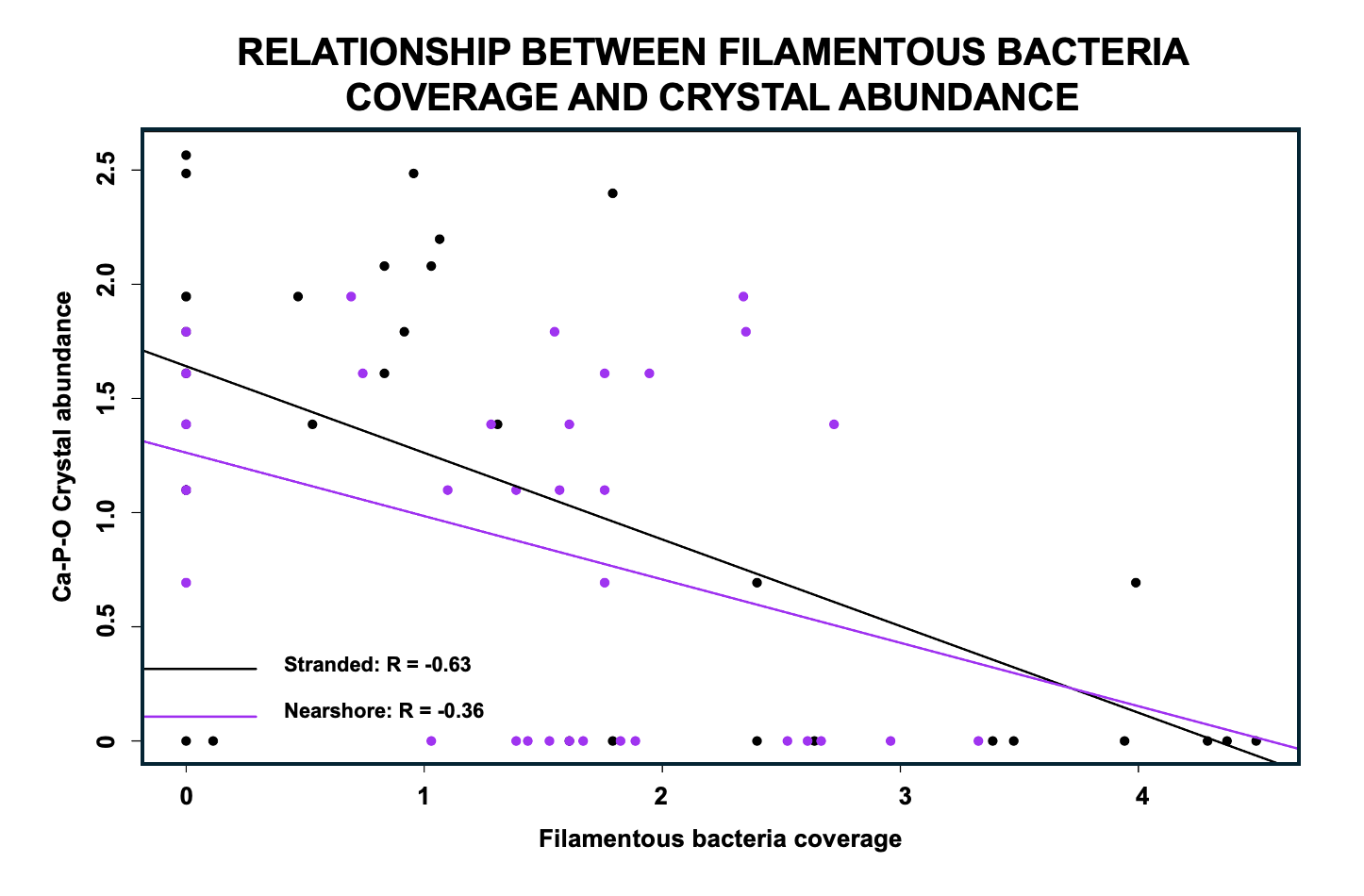
**

**Figure S8.** Correlation between log-transformed crystal abundance and log-transformed filamentous bacteria coverage in holopelagic *Sargassum (S. fluitans* var. *fluitans, S. natans v*ar. *natans and S. natans* var. *wingei)*. Data from all three thallus parts and all species were combined for each condition (stranded vs. nearshore). The reported R value represents the strength and direction of the linear relationship between the two variables at each station (Nearshore or Stranded).

**Table 1.** Parameters associated with surface roughness (contrast, entropy; blue) and surface smoothness (angular second moment, correlation, inverse difference moment) measured at four orientations (0°, 90°, 180°, 270°) in the three taxa (SFF, SNN, SNW, See Legend of Figure 1 for names of taxa) and thallus parts (axis, frond, floating vesicles or f.v.).

| **Sample** | **Angle** | **Angular Second Moment** | **Contrast** | **Correlation** | **Inverse Difference Moment** | **Entropy** |
| --- | --- | --- | --- | --- | --- | --- |
| SFF Axis | 0 | 8.401E-5 | 577.465 | 3.428E-4 | 0.058 | 9.707 |
| SFF Axis | 90 | 8.289E-5 | 584.972 | 3.421E-4 | 0.057 | 9.718 |
| SFF Axis | 180 | 8.401E-5 | 575.939 | 3.430E-4 | 0.058 | 9.707 |
| SFF Axis | 270 | 8.289E-5 | 584.010 | 3.422E-4 | 0.057 | 9.719 |
| SFF f.v. | 90 | 1.289E-4 | 491.932 | 4.202E-4 | 0.065 | 9.420 |
| SFF f.v. | 0 | 1.251E-4 | 538.659 | 4.146E-4 | 0.063 | 9.458 |
| SFF f.v. | 180 | 1.289E-4 | 490.387 | 4.204E-4 | 0.065 | 9.420 |
| SFF f.v. | 270 | 1.251E-4 | 537.847 | 4.148E-4 | 0.063 | 9.457 |
| SFF fronds | 0 | 8.813E-5 | 673.871 | 3.660E-4 | 0.049 | 9.648 |
| SFF fronds | 90 | 8.646E-5 | 654.783 | 3.633E-4 | 0.049 | 9.670 |
| SFF fronds | 180 | 8.812E-5 | 625.480 | 3.658E-4 | 0.049 | 9.648 |
| SFF fronds | 270 | 8.646E-5 | 662.835 | 3.625E-4 | 0.049 | 9.671 |
| SNN Axis | 0 | 1.733E-4 | 457.837 | 6.857E-4 | 0.065 | 9.104 |
| SNN Axis | 90 | 1.704E-4 | 483.134 | 6.762E-4 | 0.064 | 9.124 |
| SNN Axis | 180 | 1.733E-4 | 459.414 | 6.850E-4 | 0.065 | 9.104 |
| SNN Axis | 270 | 1.704E-4 | 485.029 | 6.754E-4 | 0.064 | 9.124 |
| SNN f.v. | 0 | 2.186E-4 | 235.583 | 6.523E-4 | 0.088 | 8.872 |
| SNN f.v. | 90 | 2.123E-4 | 266.369 | 6.442E-4 | 0.085 | 8.918 |
| SNN f.v. | 180 | 2.186E-4 | 238.070 | 6.516E-4 | 0.088 | 8.872 |
| SNN f.v. | 270 | 2.123E-4 | 271.083 | 6.427E-4 | 0.085 | 8.919 |
| SNN fronds | 0 | 1.243E-4 | 640.862 | 6.213E-4 | 0.052 | 9.363 |
| SNN fronds | 90 | 1.205E-4 | 702.076 | 5.984E-4 | 0.051 | 9.398 |
| SNN fronds | 180 | 1.243E-4 | 643.800 | 6.201E-4 | 0.052 | 9.364 |
| SNN fronds | 270 | 1.205E-4 | 696.932 | 6.004E-4 | 0.051 | 9.397 |
| SNW Axis | 0 | 1.215E-4 | 481.426 | 5.534E-4 | 0.062 | 9.364 |
| SNW Axis | 90 | 1.198E-4 | 488.768 | 5.520E-4 | 0.061 | 9.373 |
| SNW Axis | 180 | 1.215E-4 | 478.611 | 5.540E-4 | 0.062 | 9.364 |
| SNW Axis | 270 | 1.198E-4 | 499.053 | 5.494E-4 | 0.061 | 9.374 |
| SNW f.v. | 0 | 8.424E-5 | 550.696 | 4.068E-4 | 0.056 | 9.665 |
| SNW f.v. | 90 | 8.244E-5 | 580.442 | 4.034E-4 | 0.055 | 9.687 |
| SNW f.v. | 180 | 8.424E-5 | 553.687 | 4.065E-4 | 0.056 | 9.665 |
| SNW f.v. | 270 | 8.244E-5 | 586.500 | 4.027E-4 | 0.055 | 9.687 |
| SNW fronds | 0 | 1.015E-4 | 649.423 | 4.925E-4 | 0.051 | 9.554 |
| SNW fronds | 90 | 9.931E-5 | 695.686 | 4.833E-4 | 0.050 | 9.579 |
| SNW fronds | 180 | 1.015E-4 | 655.927 | 4.911E-4 | 0.051 | 9.554 |
| SNW fronds | 270 | 9.931E-5 | 693.226 | 4.838E-4 | 0.050 | 9.579 |
